# A Modular Customizable Ligand-Conjugate (LC) System Targeting Ghrelin *O*-Acyltransferase

**DOI:** 10.3390/biom15020204

**Published:** 2025-02-01

**Authors:** Amber L. Ford, Caine W. Taft, Andrea M. Sprague-Getsy, Gracie C. Carlson, Nilamber A. Mate, Michelle A. Sieburg, John D. Chisholm, James L. Hougland

**Affiliations:** 1 Department of Chemistry, Syracuse University, Syracuse, NY 13244, USA; alford@syr.edu (A.L.F.); cwtaft@syr.edu (C.W.T.); anspragu@syr.edu (A.M.S.-G.); gcc44@case.edu (G.C.C.); namate@syr.edu (N.A.M.); msieburg@syr.edu (M.A.S.); jdchisho@syr.edu (J.D.C.); 2 BioInspired Syracuse, Syracuse University, Syracuse, NY 13244, USA; 3 Department of Biology, Syracuse University, Syracuse, NY 13244, USA

**Keywords:** ghrelin, ghrelin *O*-acyltransferase, membrane-bound *O*-acyltransferase, GHSR, posttranslational modification, membrane enzyme, protein acylation, peptide mimetic inhibitor

## Abstract

Ghrelin is a 28 amino acid peptide hormone that impacts a wide range of biological processes, including appetite regulation, glucose metabolism, growth hormone regulation, and cognitive function. To bind and activate its cognate receptor, ghrelin must be acylated on a serine residue in a post-translational modification performed by ghrelin *O*-acyltransferase (GOAT). GOAT is a membrane-bound *O*-acyltransferase (MBOAT) responsible for the catalysis of the addition of an octanoyl fatty acid to the third serine of desacyl ghrelin. Beyond its canonical role for ghrelin maturation in endocrine cells within the stomach, GOAT was recently reported to be overexpressed in prostate cancer (PCa) cells and detected at increased levels in the serum and urine of PCa patients. This suggests GOAT can serve as a potential route for the detection and therapeutic targeting of PCa and other diseases that exhibit GOAT overexpression. Building upon a ghrelin mimetic peptide with nanomolar affinity for GOAT, we developed an antibody-conjugate-inspired system for customizable ligand-conjugate (LC) synthesis allowing for the attachment of a wide range of cargoes. The developed synthetic scheme allows for the easy synthesis of the desired LCs and demonstrates that our ligand system tolerates an extensive palette of cargoes while maintaining nanomolar affinity against GOAT.

## 1. Introduction

Ghrelin is a 28-amino-acid peptide hormone first discovered by Kojima and coworkers in 1999 [1]. Ghrelin was at first called “the hunger hormone” due to its role in appetite stimulation [2,3,4], but it has also been shown to play a role in a variety of important processes including growth hormone secretion and glucose metabolism [1,5,6,7], stress response [8,9,10], and learning and memory [11,12], and a potential role in eating disorders and addiction [13,14,15,16,17,18,19,20,21,22,23]. Its importance in these metabolic and physiological pathways makes ghrelin an attractive focus for potential therapeutic avenues for associated diseases.

Ghrelin exists in two forms in circulation: an activated form that is acylated with an octanoyl moiety of fatty acid linked to the third serine (herby called ghrelin) and a deactivated form that has a free hydroxyl at the third serine (desacyl ghrelin) [1,24,25]. Upon octanoylation, ghrelin binds its cognate receptor, the growth hormone secretagogue receptor (GHSR) [26,27,28]. GHSR is a G protein-coupled receptor (GPCR) that, when activated by ghrelin, initiates several signaling cascades [7,29]. The unique covalent addition of the octanoyl chain to ghrelin is catalyzed by the enzyme ghrelin *O*-acyltransferase (GOAT) [30,31,32]. GOAT was originally discovered in stomach tissue [1]; however, several studies have reported GOAT expression in a variety of tissues [31,33]. Notably, recent studies have reported the overexpression of GOAT in several cancers, including prostate cancer and breast cancers [34,35,36,37,38]. These cancer cells are also reported to express ghrelin, which may act within a noncanonical pathway in these cells [37,39,40,41,42,43]. Furthermore, there is evidence supporting GOAT expression at the plasma membrane and in extracellular vesicles, which expands upon its canonical localization in the endoplasmic reticulum [44,45,46,47,48]. Since GOAT is found to be overexpressed in some cancerous tissues [34,37], this makes GOAT an attractive target for potential diagnostic and therapeutic development.

To detect GOAT in biological systems, we developed a ghrelin-mimetic peptide ligand with a high affinity and specificity for GOAT without off-target binding to GHSR. Conjugating a fluorophore to this peptide ligand had minimal effect on the binding affinity to GOAT [44,49]. This success in adding cargo to the original ligand inspired further exploration of ligand conjugation to develop a ligand-conjugate (LC) system targeting GOAT. LC systems can provide an economical alternative approach to antibody–drug conjugates (ADCs) currently being explored as anticancer agents [50,51,52,53]. ADCs can provide cancer-specific treatment with a large therapeutic payload and minimal side effects [54]. These drugs have been shown in prostate cancer patients and in clinical trials to be effective as diagnostics and treatments [55,56,57,58]. While customizable and effective in treating cancer, ADCs are also expensive and difficult to synthesize [54]. Due to the rigor required to synthesize ADCs and the lack of ADCs for prostate cancer, we propose using a peptide-based approach to target GOAT to develop new cancer diagnostics or therapeutics. This approach may mitigate the expense and difficulty associated with synthesizing ADCs, replacing them with a lower-cost system.

Toward this goal, we have developed a customizable system for developing GOAT-targeted ligand conjugates (LCs) using a parent peptide sequence with a high affinity and specificity for GOAT, a novel bifunctional linker molecule, and an array of cargo molecules. Using this modular synthetic scheme, we developed a library of GOAT-specific ligands with different cargoes. The ligand characterization demonstrated a broad tolerance to the cargo attachment without the loss of binding affinity to GOAT, although the linker composition can negatively impact the ligand potency. This customizable system for generating potent GOAT-specific ligands may be used in the future for prostate cancer detection and treatment.

## 2. Materials and Methods

### 2.1. General

All of the parent peptides (**3**, **3**′, **4**, **16**, and **16′**) were commercially available from BioBasic (Markham, ON, Canada) and Pepmic (Suzhou, China). The peptides were solubilized in 50% aqueous acetonitrile and ultrapure water at 5 mM (by peptide mass) for testing and storage at −20 °C. Following synthesis, the bifunctional adapter was solubilized in 50% aqueous acetonitrile at 20 mM and aliquoted for storage at −20 °C. The TAMRA azide was purchased from Click Chemistry Tools (Scottsdale, AZ, USA) and solubilized at a 10 mM concentration in 500 µL of DMSO for storage while protected from light at −20 °C. The fluorophores AF488 alkyne, AF488 azide, FAM5 azide, SulfoCy5 azide, and SulfoCy5 maleimide were obtained from Lumiprobe (Hunt Valley, MD, USA) and solubilized in anhydrous DMSO in 10 mM stocks and stored at −20 °C, protected from light. The quenchers TideQuencher2, AzoDye1 alkyne, and AzoDye1 azide were obtained from AAT Bioquest (Pleasonton, CA, USA) and solubilized in anhydrous DMSO in 1 mM stocks and stored at −20 °C, protected from light. The mertansine was obtained from Cayman Chemical (Ann Arbor, MI, USA) and was solubilized in DMSO in 20 mM aliquots stored at −20 °C. The methyl arachidonyl fluorophosphonate (MAFP) was diluted in DMSO from a stock in methyl acetate obtained from Cayman Chemical (Ann Arbor, MI, USA). The octanoyl coenzyme A (octanoyl CoA, free acid) was purchased from AdventBio (Elk Grove Village, IL, USA) and was solubilized to 5 mM in 10 mM Tris-HCL pH 7.0 and stored in low-adhesion tubes at −80 °C until use. The GSSFLC_NH2_ peptide used in the fluorescent acrylodan labeling was obtained from Sigma-Genosys (The Woodlands, TX, USA) and synthesized in Pepscreen format. The GSSFLC_NH2_ peptide was solubilized in 100% acetonitrile and stored at −80 °C. The acrylodan was obtained from Fisher Scientific (Waltham, MA, USA) and solubilized in 100% acetonitrile in 2.2 mM stocks for storage at −80 °C and protected from light until use. The copper (I) iodide, copper (II) sulfate, and sodium ascorbate were obtained from Sigma Aldrich (Burlington, MA, USA) and stored at room temperature. The tris(3-hydroxypropyltriazolylmethyl) amine (THPTA) was obtained from Lumiprobe (Hunt Valley, MD, USA) and stored at −20 °C, protected from light. The MALDI matrix α-Cyano-4-hydroxycinnamic acid (HCCA, Sigma Aldrich, Darmstadt, Germany) was stored at room temperature. The stocks were created as indicated in the MALDI sample preparation section and stored at room temperature until use. Fresh stocks were created every six months.

### 2.2. Peptide Ligand Concentration Determination

The peptide ligand concentrations were determined by UV-Vis absorbance using various chromophores. For the peptides lacking a fluorophore or quencher cargo, the concentrations were determined using absorbance of the unnatural amino acid 1-naphthylalanine (Nal) at 280 nm (ε_280_ = 3936.12 M^−1^ cm^−1^) [46,59] The concentrations for ligands containing fluorophores were determined using fluorophore absorbance as follows: TAMRA, absorbance at 553 nm (λ_ex_ = 553 nm, λ_em_ = 575 nm, ε_553_ = 92,000 M^−1^ cm^−1^, per the manufacturer); FAM5, absorbance at 492 nm (λ_ex_ = 492 nm, λ_em_ = 517 nm, ε_492_ = 74,000 M^−1^ cm^−1^, per the manufacturer); AF488, absorbance at 495 nm (λ_ex_ = 495 nm, λ_em_ = 519 nm, ε_495_ = 71,800 M^−1^ cm^−1^, per the manufacturer); and SulfoCy5, absorbance at 646 nm (λ_ex_ = 646 nm, λ_em_ = 662 nm, ε_646_ = 271,000 M^−1^ cm^−1^, per the manufacturer). The concentrations for the ligands containing quenchers were determined using quencher absorbance as follows: AzoDye1 azide, 522 nm (ε_522_ = 34,000 M^−1^ cm^−1^); AzoDye1 alkyne, 522 nm (ε_522_ = 34,000 M^−1^ cm^−1^); and TideQuencher2 azide, 540 nm (ε_540_ = 21,000 M^−1^ cm^−1^).

### 2.3. Bifunctional Adapter Synthesis

The bifunctional adapter was synthesized from the corresponding diol precursor in two steps, with the first step following published procedure [60]. Details of the synthetic protocols and the compound characterizations are reported in the Supplementary Material and in Supplemental Figures S1–S4. 

### 2.4. hGOAT Channel Computational Analysis

The distance from the putative active site/ligand-binding site to the surface of the hGOAT was determined using the computational model of hGOAT, with the distances analyzed using the PyMOL Molecular Graphics System, Version 2.5.3 from Shrödinger, LLC (New York, NY, USA). The average distances between the residue His338 and the residues lining the luminal pore were measured using the wizard measurement tool. For the computational model of hGOAT, residues M1, E105, l232, and S401 were selected based on their proximity to the entrance of the transmembrane channel. The distance from His338, atom NE2 was calculated to atom SD on M1, atom CD on E105, atom CG on L232, and atom OG on S401. The images of the model were all created within the PyMOL Molecular Graphics System, Version 2.5.3 from Shrödinger, LLC.

### 2.5. Copper-Catalyzed Cycloaddition for Cargo Attachment

#### 2.5.1. Cu(I)I Azide–Alkyne Cycloaddition

For the majority of the copper-catalyzed cycloadditions of the azidohomoalanine- or azide-bearing cargoes and alkynes, a literature procedure was followed [61]. The standard protocol combined 3.3 mg (0.017 mmol) Cu(I)I and 7 mg (0.013) mmol TBTA in 500 µL 4:1 DMF:H2O in a 5 mL test tube equipped with a stir bar. The mixture was allowed to stir for approximately 15 min until the reaction turned a pale yellow color. For the conjugation of the parent AHA peptide (azide) and the bifunctional linker (alkyne), after mixing, 0.0004 mmol (160 µL, 5 mM stock) of the parent peptide was added along with 0.004 mmol (2.1 mg) of the bifunctional linker. For the conjugation of the AHA–linker conjugate (alkyne) and the TAMRA azide (azide), after mixing, 0.0004 mmol of the parent peptide or the peptide–linker conjugate was added along with 0.004 mmol of the cargo, with the volumes varying depending on the concentration. This protocol was halved or doubled based on the amount of the starting material available. The reaction was allowed to stir vigorously for approximately 18 h at room temperature. The final reaction turned a dark brown–yellow color. The reaction was then transferred to a 1.5 mL Eppendorf tube and centrifuged at the maximum speed for 10 min to prepare for the HPLC purification, as described below.

#### 2.5.2. Cu(II)SO_4_ Azide–Alkyne Cycloaddition

The protocol followed for this method of azide–alkyne cycloaddition was modeled after a reported protocol optimized by the manufacturer, BroadPharm [62]. A total of 200 mM tris-hydroxypropyltriazolylmethylamine (THTPA), 100 mM copper sulfate pentahydrate (CuSO_4_-5H_2_O), and 100 mM sodium ascorbate was prepared in UP H_2_O prior to the reaction. The copper:THPTA complex was formed by creating a 1:1 mixture of THPTA and CuSO_4_-5H_2_O (in a 1:2 ratio of CuSO_4_-5H_2_O:THPTA) and allowing it to incubate for 15 min until a dark blue color appeared. After the complex formation, 37.5 µL of the complex (7.5 and 15 µM concentrations), 12 mM sodium ascorbate, 300 µM Dap-AHA, and 1.2 mM AF488 alkyne were combined in a black 1.5 mL Eppendorf tube and allowed to stand for 1 h at room temperature. The semipreparative HPLC purification of the product was achieved using the method described in the next section.

### 2.6. Thiol–Maleimide Conjugation for Cargo Attachment

For the thiol–maleimide conjugations, 300 µM of the peptide (parent or parent–linker conjugate) and 500 µM of the bifunctional linker or cargo (mertansine) were combined in 50 mM HEPES buffer pH 7.8 and 50% *v*/*v* acetonitrile in a total volume of 500 µL. The conjugation of the AHA–linker conjugate with the mertansine required the dropwise addition of the mertansine to prevent disulfide formation. The conjugation reactions were vortexed for approximately 16 h at room temperature at mid-speed. The vortexing was achieved using a Vortex Genie 2 from Scientific Industries (Bohemia, NY, USA). The reactions were purified by HPLC as described below.

### 2.7. Purification of LCs via Semipreparative HPLC

The peptide conjugation reactions were purified by semipreparative reverse-phase HPLC (Zorbax Eclipse XDB-C18 column, 9.4 × 250 mm) using a solvent gradient from 2% acetonitrile in 0.05% aqueous TFA to 98% acetonitrile in 0.05% aqueous TFA over 35 min at a flow rate of 4.2 mL/min, followed by 100% acetonitrile for 10 min. The total time for each run was 45 min. The peptides and conjugates were detected by UV absorbance at 280 nm by the unnatural amino acid 1-naphthylalanine, and the peptides were collected in 1.5 mL Eppendorf tubes. The retention times of all LCs are reported in Supplemental Table S1. Chemstation for LC (Agilent Technologies, Santa Clara, CA, USA) was used for peak integration to determine purity. All of the samples were dried by SpeedVac (Eppendorf Vacufuge plus, Enfield, CT, USA) for 10 h under vacuum. The dried peptides and LCs were redissolved in 30–60 µL of 50% aqueous acetonitrile. The concentrations were determined by UV-visible absorbance (ThermoFisher scientific nanodrop 2000c spectrophotometer, Thermo Fisher Scientific Inc, Waltham, MA, USA) at the specified wavelength as described above. All of the stocks were stored at −20 °C.

### 2.8. MALDI Characterization of LCs

The LCs were characterized by MALDI mass spectrometry using an α-cyano-4-hydroxycinnamic acid (CHCA, Sigma Aldrich, Darmstadt, Germany) matrix. The samples were analyzed by MALDI-TOF Microflex LRF (Bruker, Billerica, MA, USA) using the RP 500–3500 Da method. The MALDI spectra were analyzed by FlexAnalysis software Version 3.4. The masses for all LCs are reported in Supplementary Table S1.

### 2.9. IC_50_ Protocol

The microsomal fraction containing hGOAT for the ligand-binding studies by IC_50_ determination was prepared according to published protocols [63]. For the GOAT inhibition assay, serial dilutions of 0, 0.15, 0.5, 1, 5, 15, and 50 µM concentrations were prepared in 50% acetonitrile for each ligand used. The membrane fractions from the Sf9 cells expressing GOAT were first thawed on ice then passed through an 18-gauge needle 10 times to homogenize them. The assays were performed with approximately 30 µg of the membrane protein as determined through a Bradford assay. The standard reaction conditions contained 50 mM HEPES pH 7.0, 1 µM MAFP, 30 µg of membrane protein, and ultrapure water. The ligands were added to the reaction mixtures and incubated for 30 min at room temperature protected from light prior to the reaction initiation. The reactions were initiated by octanoyl CoA (300 µM final concentration) and GSSFLC_Acdan_ (0.3 µM final concentration) in a total reaction volume of 50 µL. The reactions were incubated for 45–120 min protected from light at room temperature until 30–50% substrate acylation was observed in the vehicle control. The reactions were stopped with 50 µL of 20% acetic acid in isopropanol. The excess membrane fraction was precipitated with 16.7 µL of 40% trichloroacetic acid (TCA) followed by a 1000× *g* centrifugation for 2 min. The supernatant was then analyzed via reverse-phase HPLC in 100 µL samples as previously described [63]. The inhibition trials were run in triplicate, with the reported IC_50_ values representing the average of at least three trials. The IC_50_ values for all LCs are reported in Supplemental Table S2.

### 2.10. Analysis of GOAT Inhibition Assays via Analytical HPLC

The assays were analyzed on reverse-phase HPLC (Zorbax Eclipse XDB column, 4.6 × 150 mm) using a solvent gradient from 100% acetonitrile to 63% acetonitrile in aqueous 0.05% TFA over 5 min at a flow rate of 1 mL/min, followed by a gradient of 100% acetonitrile in 0.05% TFA to 63% acetonitrile in 0.05% TFA over 1 min, 100% acetonitrile for 5 min, and a gradient of 100% acetonitrile to 30% acetonitrile in 0.05% TFA for 5 min. The total time for each run was 16 min. The peptides were detected by the attached acrylodan label with the UV absorbance at 360 nm and fluorescence (λ_ex_ = 360 nm, λ_em_ = 485 nm). The octanoylated peptide typically eluted at around 7.5 min, with the unreacted peptide eluting at around 2.5 min. Chemstation for LC (Agilent Technologies) was used for peak integration.

### 2.11. Determination of IC_50_ Values

The peak integrations were used to determine the percent conversion in the presence of either the inhibitor (ligand, LC) or the vehicle (50% acetonitrile). The percent activity was calculated using Equation (1):

(1) $$\%\;peptide\;octanoylationa=\frac{fluorescence\;of\;octanoylated\;peptide}{total\;peptide\;fluorescence},$$

IC_50_ values were determined by fitting Equation (2) to the plot of % activity against inhibitor concentration:

(2) $$\%\;activity=\%\;vehicle\left(1-\frac{\left[Inhibitor\right]}{\left[Inhibitor\right]+IC50}\right)$$

The plots and data fitting were performed with Kaleidagraph (Synergy Software Version 4.1, Reading, PA, USA).

## 3. Results and Discussion

### 3.1. Design of Modular Ligand-Conjugate System

**Figure 1:**
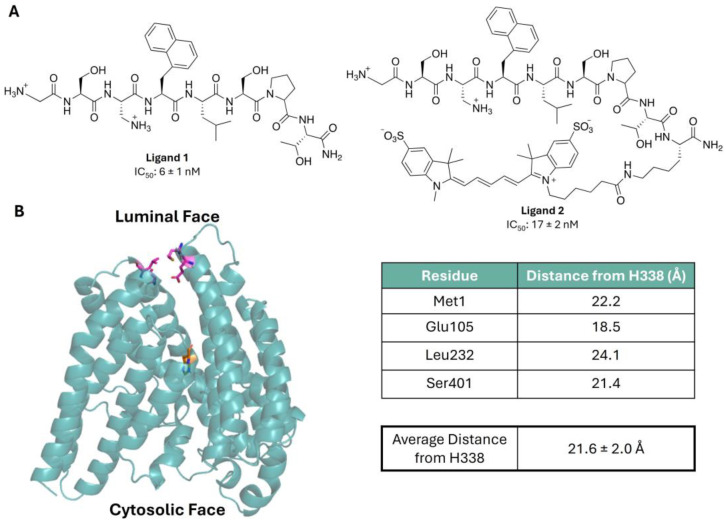
Initial ligands and constraints for a new generation of customizable GOAT ligands. (**A**) The structures of GOAT ligand **1** and its fluorophore-conjugated derivative ligand **2** [44]. (**B**) The computational model of human GOAT (hGOAT) depicting the distance from the conserved catalytically essential H338 residue to multiple residues residing on the luminal pore through which ghrelin is proposed to enter the enzyme catalytic transmembrane channel. The distances for each pore residue are measured from the H338 side chain as described in the Materials and Methods Section. hGOAT is shown in dark teal, H338 is shown in orange, and selected pore-defining residues (Met1, Glu105, Leu232, and Ser401) are shown in magenta. Images of the hGOAT computational model was created in the PyMOL Molecular Graphics System.

We previously reported the development of a ghrelin mimetic ligand (**1**) with a high specificity and affinity for GOAT based on structure–activity relationships, and further modification yielded the second-generation ligand with a fluorescent SulfoCy5 tag (**2**) (Figure 1A) [49]. The attachment of this fluorophore yields a small three-fold loss of binding potency to GOAT. To reduce the likelihood of cargo interaction with GOAT in our ligand-conjugate library, we examined the structural model of human GOAT to determine the optimal ligand and linker lengths. In this analysis, we leveraged our finding that the interaction between the Dap amino side chain at the third position of the peptide ligand and the conserved H338 residue within the GOAT catalytic channel is essential for ligand binding [44]. This catalytic channel, which is a common feature of protein-modifying MBOAT family members [64,65,66,67], connects the pore through which ghrelin enters the core of GOAT with the acyl-donor-binding site at the enzyme’s cytoplasmic face [46]. The side chain of H338 lies ~21 Å from the pore interface, as measured to four GOAT residues on the pore periphery (Figure 1B). With five amino acids between the Dap residue contacting H338 and the fluorophore attachment point, this distance is compatible with the fluorophore in ligand **2** lying outside the GOAT channel and exposed to solvent [68,69]. However, in our customizable ligand-conjugate system, we chose to add additional spacing between the GOAT ligand and the cargo to further reduce any chance of direct cargo contact with GOAT or its proximal membrane lipids.

**Figure 2:**
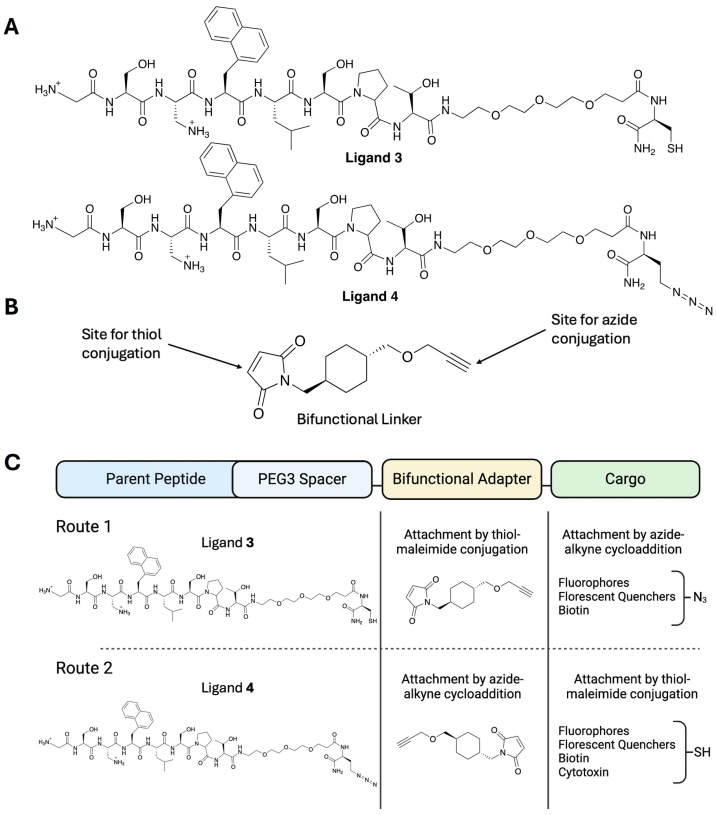
The design concept for the GOAT ligand-conjugate (LC) system. (**A**) The structures of parent ligands **3** and **4**, based on ligand **1** with the PEG linker and the chemospecific thiol or azide conjugation tags. (**B**) The structure of the bifunctional linker molecule. (**C**) The scheme for the construction of the customizable LC library including the base ligand, PEG3 spacer, bifunctional adapter, and cargo.

For the GOAT peptide ligand in this ligand conjugate system, we designed two parent peptides predicted to maintain nanomolar affinity for GOAT while allowing for ample customization at their C-termini. These peptides share the same eight-residue ghrelin–mimetic sequence as the previously developed ligands, followed by a mini-PEG3 linker terminating with either a C-amidated cysteine residue (**3**) for the thiol–maleimide conjugation or C-amidated azidohomoalanine (**4**) for the azide–alkyne copper-catalyzed cycloaddition (Figure 2A, Supplemental Figures S5 and S6). To provide further spacing between the peptide ligand and the cargo and allow for additional conjugation flexibility, we developed a novel bifunctional linker molecule containing maleimide and alkyne sites for cargo attachment using thiol–maleimide or azide–alkyne conjugation chemistries (Figure 2B). This bifunctional linker was inspired by the bifunctional maleimide–NHS ester linker used in trastuzumab–emtansine, which is an FDA-approved ADC for the treatment of HER2-positive breast cancer [70]. Our scheme combines these modular components with the desired cargo molecules in a customizable method for LC development (Figure 2C).

### 3.2. Ligand Synthesis and Characterization of GOAT-Binding Potency

**Scheme 1:**
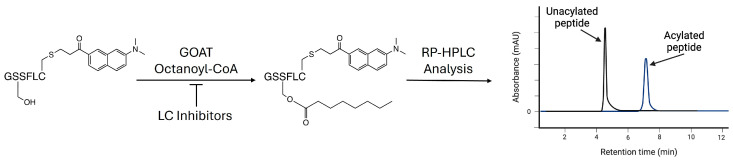
The GOAT inhibition assay for determining ligand binding. The octanoylation of a fluorescently labeled ghrelin-mimetic peptide substrate is inhibited by LC binding, with GOAT octanoylation activity and inhibition determined as described in the Section 2.

**Figure 3:**
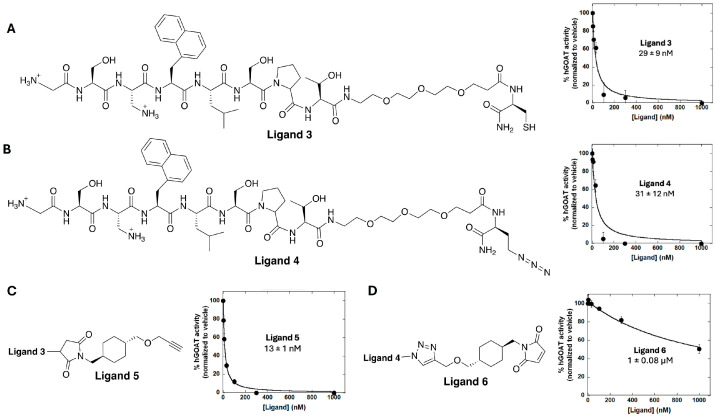
The parent ligand and ligand–linker conjugate binding to hGOAT. (**A**) The structure and IC_50_ determination of ligand **3** terminating in a cysteine residue. (**B**) The structure and IC_50_ determination of ligand **4** terminating in an azidohomoalanine (Aha) residue. (**C**) The abbreviated structure and IC_50_ determination of ligand **5**. (**D**) The abbreviated structure and IC_50_ determination of ligand **6**. The IC_50_ values against hGOAT represent the average of three independent trials, and the error bars represent one standard deviation.

The binding potency to GOAT for all of the ligands and ligand conjugates was determined using our standard GOAT ghrelin acylation inhibition assay (Scheme 1) [63,71]. Both parent peptides **3** and **4** exhibited a five-fold decrease in GOAT affinity compared to the first-generation ligand **1** and a two-fold decrease in GOAT affinity from the second-generation fluorescent ligand **2** but still maintained nanomolar affinity for GOAT (Figure 3A,B). These ligands can be used to directly attach to the cargo of choice using their thiol and azide moieties if rigidity or proximity to GOAT are warranted, or they can be further functionalized using the novel bifunctional linker molecule to introduce thiol- and azide-reactive attachment points. Ligand **3** was conjugated to the bifunctional linker using a thiol–maleimide addition to yield ligand **5**. Ligand **5** exhibits increased GOAT binding affinity with potency comparable to ligand **2** (Figure 1A and Figure 3C, Supplemental Figure S7). The attachment of the bifunctional linker molecule to parent ligand **4** was achieved using a copper-catalyzed azide–alkyne cycloaddition. This yielded ligand **6**, which exhibited a drastic decrease in the apparent GOAT inhibition potency (Figure 3D, Supplemental Figure S8). This loss of potency may reflect the sequestration of ligand **6** by the reaction of the free maleimide–thiol groups on the background proteins in the membrane protein fractions used in our GOAT acylation assays [63]. If this maleimide side reactivity is the cause of the diminished binding for ligand **6**, we predict this effect will be alleviated by the addition of a thiol-bearing cargo to ligand **6**, which will functionalize the maleimide group.

### 3.3. Exploring the Scope of Potential Cargo Molecules for GOAT Ligand-Conjugates

**Figure 4:**
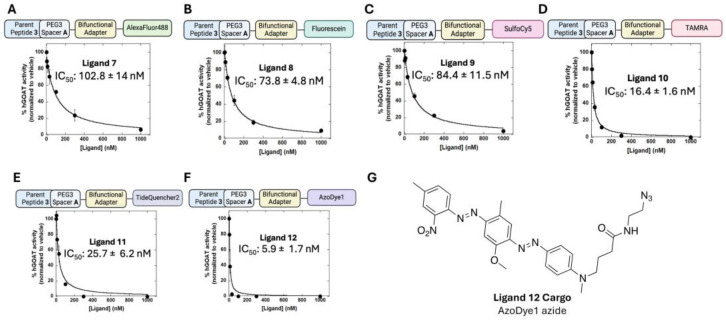
The assessment of cargo tolerance in the hGOAT LC system. (**A**–**D**) The IC_50_ values for fluorescently conjugated ligands **7**, **8**, **9**, and **10** with AF488, fluoresceine, SulfoCy5, and TAMRA cargoes, respectively. (**E**,**F**) The IC_50_ values for ligands **11** and **12** with fluorescence quencher cargoes. (**G**) The structure of the fluorescence quenching cargo AzoDye1 azide (ligand **12** cargo). The IC_50_ values against hGOAT represent the average of three independent trials, and the error bars represent one standard deviation.

Building upon ligand **5**, we determined the impact of cargo conjugation on the GOAT-binding potency. The first cargoes we explored were fluorophores to enable GOAT detection/labeling and ligand-binding studies [44]. The fluorophores chosen covered a range of excitation and emission wavelengths as well as differing chemical properties (e.g., size and charge) to assess the diversity of the cargoes tolerated in this ligand-conjugate system. Fluorophore azides were conjugated to ligand **5** to create ligands **7** (AF488 azide), **8** (FAM5 azide), **9** (SulfoCy5 azide), and **10** (TAMRA azide). The IC_50_ values for ligands **7**, **8**, and **9** are six- to eight-fold higher than that of parent ligand **5**, with the AF488 ligand highest at ~100 nM (Figure 4A–C, Supplemental Figures S9–S12). Conversely, ligand **10** maintained the low IC_50_ value seen with ligand **5**, supporting that the attachment of cargo can result in little or no change to the GOAT affinity (Figure 4D). The TAMRA fluorophore in ligand **10** has a positive charge, which could contribute to maintaining the high binding affinity. Notably, all of the fluorophore-conjugated ligands maintained sub-micromolar affinity well below the affinity for native ghrelin, demonstrating the tolerance of our GOAT ligand-conjugate system for different cargoes [32,49].

We also explored LCs functionalized with fluorescent quencher cargoes to be used in fluorescence resonance energy transfer (FRET) experiments, resulting in ligands **11** and **12** (Figure 4E,F, Supplemental Figures S13 and S14). Ligand **11**, bearing a TideQuencher2 cargo, exhibited an IC_50_ approximately two-fold higher than that of the parent ligand **5**. However, ligand **12**, bearing an AzoDye1 cargo, showed a drastic increase in GOAT-binding potency to become the highest-affinity GOAT-targeting peptide-based LC reported (Figure 4F). This increase in binding potency may be due to the linear nature of the AzoDye1 fluorescent quencher combined with the neutral charge and aromatic ring system of this cargo (Figure 4G). 

**Figure 5:**
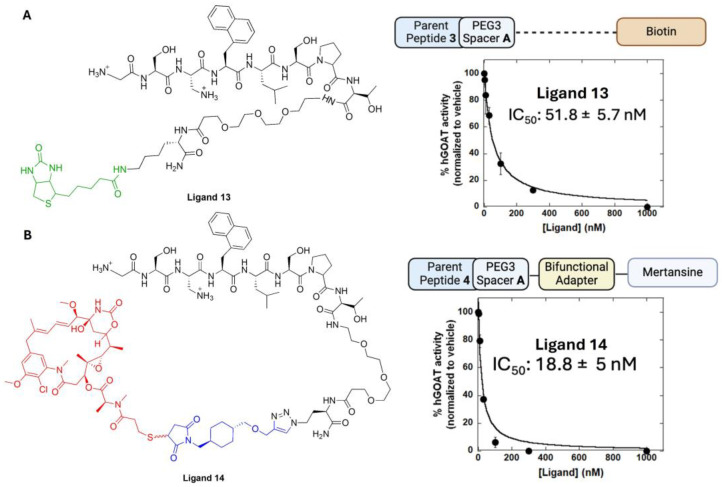
GOAT ligand conjugates bearing affinity and cytotoxin cargoes effectively bind hGOAT. (**A**) Ligand **13**, containing a biotin cargo, binds potently to hGOAT. (**B**) Ligand **14**, containing a mertansine cytotoxin cargo, exhibits strong binding to hGOAT. The IC_50_ values against hGOAT represent the average of three independent trials, and the error bars represent one standard deviation.

Complementary to the fluorescent and quencher cargoes above, we found that the attachment of cargoes for affinity labeling or potential therapeutic applications was similarly well-tolerated by GOAT. Ligand **13** contains a biotin at the ligand C-terminal end to enable detection and isolation by streptavidin binding. This ligand did not include the bifunctional adapter and exhibited GOAT-binding affinity within two-fold of the comparable parent peptide ligand **3** (Figure 5A, Supplemental Figure S15). Ligand **14** bears the cytotoxin mertansine attached to ligand **6** through thiol–maleimide conjugation, with this LC modeled after an FDA-approved ADC cancer treatment for breast cancer (Figure 5B, Supplemental Figure S16) [72]. Ligand **14** notably exhibits exceptional hGOAT-binding affinity (~20 nM) compared to the >1 µM apparent IC_50_ observed with ligand **6**. This is consistent with our hypothesis that the aberrant ligand **6** potency was due to off-target reactions with thiols in the membrane protein fraction. Furthermore, the attachment of the large mertansine cargo without the loss of binding potency further demonstrates the broad tolerance of our GOAT ligand conjugate system for a wide range of cargo molecules.

### 3.4. Determining the Impact of Linker Groups on GOAT Ligand Conjugate Binding

**Figure 6:**
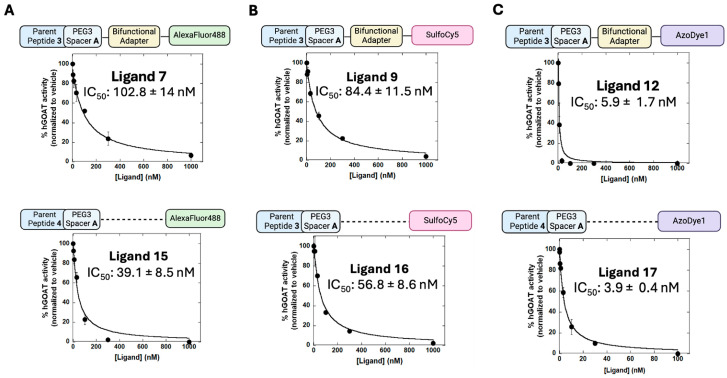
Determination of the impact of the bifunctional adapter molecule on hGOAT LC binding. The impact of the bifunctional adapter was determined by the comparison of LCs bearing the same cargo with and without the adapter. (**A**) AF488 fluorophore cargo; comparison of ligand **7** (with adapter) and ligand **15** (without adapter). (**B**) SulfoCy5 fluorophore cargo; comparison of ligand **9** (with adapter) and ligand **16** (without adapter). (**C**) AzoDye1 quencher cargo; comparison of ligand **12** (with adapter) and ligand **17** (without adapter). The IC_50_ values against hGOAT represent the average of three independent trials, and the error bars represent one standard deviation.

Having established that our ligand conjugate system readily accepts a range of cargo molecules, we determined the importance of the two linker groups in our ligand design—the bifunctional adapter and the PEG3 linker connecting the core ghrelin ligand peptide to the C-terminal cysteine or the azidohomoalanine residue. The attachment of the AF488, SulfoCy5, and AzoDye1 cargoes directly to ligand **4** by azide–alkyne cycloaddition yielded ligands **15**, **16**, and **17**, respectively (Figure 6, Supplemental Figures S17–S19). In each case, the ligand without the bifunctional linker bound GOAT more tightly, although this effect was less than three-fold in all cases. This suggests the bifunctional adapter is not required to provide a rigid linker to separate the cargo from GOAT, but in future ligand development, the incorporation of the bifunctional adapter can be explored (particularly in the case of larger cargo molecules) without concern about a substantial loss of binding potency to GOAT.

**Figure 7:**
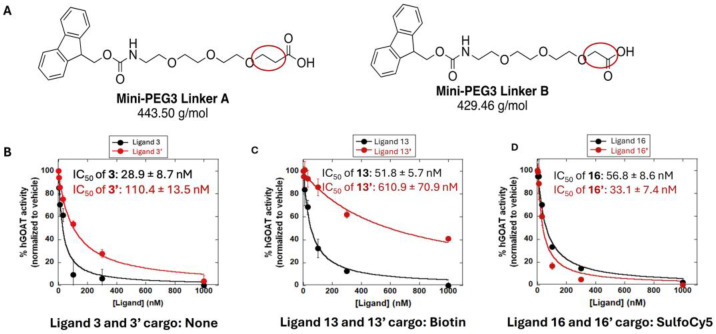
Mini-PEG3 linker composition can unexpectedly impact LC binding to hGOAT. (**A**) The structures of mini-PEG3 linkers A (443.50 g/mol) and B (429.46 g/mol) used in commercially synthesized parent ligands and subsequently derived LCs. The linkers differ by a single methylene group (red oval), which lies proximal to the cysteine or AHA cargo conjugation site. The LCs noted by a prime symbol indicate the presence of mini-PEG3 linker B. (**B**) Overlay of IC_50_ curves for ligands **3** (mini-PEG3 linker A) and **3′** (mini-PEG3 linker B). (**C**) Overlay of IC_50_ curves for ligands **13** (mini-PEG3 linker A) and **13′** (mini-PEG3 linker B). (**D**) Overlay of IC_50_ curves for ligands **16** (mini-PEG3 linker A) and **16′** (mini-PEG3 linker B). The IC_50_ values against hGOAT represent the average of three independent trials, and the error bars represent one standard deviation.

During the preparation of our ligand conjugate library, we discovered that the “mini-PEG3” linker offered by different commercial suppliers can vary in structure, with either one or two methylene carbons between the carboxylate terminus and the first ether oxygen (Figure 7A). To determine if this change in linker structure could impact ligand binding to GOAT, we prepared ligands corresponding to ligands **3**, **13**, and **16** bearing the one-methylene version of the PEG3 linker (denoted linker B) (Figure 7B–D, Supplemental Figures S20–S22). In two of three cases, the incorporation of the PEG3 linker B, with the loss of a single methylene unit in the spacer, resulted in a loss of binding potency with an approximately four-fold drop for the parent peptide (**3** and **3′**) and an approximately seven-fold loss for the biotin-bearing ligand (**13** and **13′**). In contrast, the ligand bearing the Sulfo-Cy5 fluorophore with the PEG3 linker B exhibited an approximately two-fold increase in binding affinity to GOAT compared to linker A (**16** and **16′**). The decrease in the IC_50_ value in this case may reflect the specific properties of the sulfoCy5 fluorophore and an interaction with either the GOAT or the surrounding membrane phospholipids. SulfoCy5 was the only negatively charged cargo evaluated in this LC library, and notably the attachment of SulfoCy5 to each ligand in this study decreased the GOAT-binding affinity by at least two-fold, other than ligand **16′** with the shortened PEG3 linker. These results highlight the structure of the mini-PEG3 linker as a potential variable to be considered in ligand design and optimization.

## 4. Conclusions

We have a demonstrated an efficient route toward the creation of a customizable LC library for GOAT. This library allows for two orthogonal modes for cargo attachment, a maleimide–thiol conjugation and an azide–alkyne copper catalyzed cycloaddition. This synthesis is robust and compatible with a variety of cargo molecules differing in size and charge. These results indicate that while cargo size, flexibility, and charge can affect binding affinity to GOAT, overall this LC system can accommodate a variety of cargoes without losing potency against GOAT. These LCs will enable new studies of GOAT expression in cells and tissues and will support the exploration of GOAT as a biomarker and therapeutic target.

## Data Availability

The data and original contributions presented in this study are included in the article and Supplementary Material. Further inquiries can be directed to the corresponding author.

## Supplementary Materials

The following supporting information can be downloaded at: https://www.mdpi.com/article/10.3390/biom15020204/s1, Supporting synthetic methods; Table S1. Analytical data for peptide ligands; Table S2. Ligand-conjugate GOAT inhibitory activity; Figure S1. ^1^H NMR spectrum for compound S1; Figure S2. ^13^C NMR spectrum for compound S1; Figure S3. ^1^H NMR spectrum for compound S2; Figure S4. ^13^C NMR spectrum for compound S2; Figure S5. Characterization information for Ligand 3; Figure S6. Characterization information for Ligand 4; Figure S7. Characterization information for Ligand 5; Figure S8. Characterization information for Ligand 6; Figure S9. Characterization information for Ligand 7; Figure S10. Characterization information for Ligand 8; Figure S11. Characterization information for Ligand 9; Figure S12. Characterization information for Ligand 10; Figure S13. Characterization information for Ligand 11; Figure S14. Characterization information for Ligand 12; Figure S15. Characterization information for Ligand 13; Figure S16. Characterization information for Ligand 14; Figure S17. Characterization information for Ligand 15; Figure S18. Characterization information for Ligand 16; Figure S19. Characterization information for Ligand 17; Figure S20. Characterization information for Ligand 3’; Figure S21. Characterization information for Ligand 13’; Figure S22. Characterization information for Ligand 16’; Supplementary References.
