# Supplementary material for: A Modular Customizable Ligand-Conjugate (LC) System Targeting Ghrelin O-Acyltransferase
Source: Biomolecules. 2025 Feb 1;15(2):204. doi: 10.3390/biom15020204 (PMC11852496; doi:10.3390/biom15020204)
Supplement: Supplementary file 1 [file biomolecules-15-00204-s001.zip › biomolecules-3377899-supplementary.pdf]

Supplementary Material

Development of a customizable ligand-conjugate (LC) library targeting ghrelin *O*-acyltransferase

Amber L. Ford[1], Caine W. Taft[1], Gracie C. Carlson[1], Nilamber Mate[1], Michelle A. Sieburg[1], John Chisholm[1,2], and James L. Hougland[1,2,3]

[1]Department of Chemistry, Syracuse University, Syracuse, NY 13244, USA

[2]BioInspired Syracuse, Syracuse University, Syracuse, NY 13244, USA

[3]Department of Biology, Syracuse University, Syracuse, NY 13244, USA

James L. Hougland

Departments of Chemistry and Biology

Syracuse University

1-133 Center for Science and Technology

Syracuse, NY, 13244, USA

Tel: (315)-443-1134

Fax: (315)-443-4070

Email: hougland@syr.edu

Supporting synthetic methods

Table S1. Analytical data for peptide ligands

Table S2. Ligand-conjugate GOAT inhibitory activity

Figure S1. Characterization information for Ligand 3

Figure S2. Characterization information for Ligand 3'

Figure S3. Characterization information for Ligand 4

Figure S4. Characterization information for Ligand 5

Figure S5. Characterization information for Ligand 6

Figure S6. Characterization information for Ligand 7

Figure S7. Characterization information for Ligand 8

Figure S8. Characterization information for Ligand 9

Figure S9. Characterization information for Ligand 10

Figure S10. Characterization information for Ligand 11

Figure S11. Characterization information for Ligand 12

Figure S12. Characterization information for Ligand 13

Figure S13. Characterization information for Ligand 13'

Figure S14. Characterization information for Ligand 14

Figure S15. Characterization information for Ligand 15

Figure S16. Characterization information for Ligand 16

Figure S17. Characterization information for Ligand 16'

Figure S18. Characterization information for Ligand 17

Figure S19. ^{1}H NMR spectrum for compound S1

Figure S20. ^{13}C NMR spectrum for compound S1

Figure S21. ^{1}H NMR spectrum for compound S2

Figure S22. ^{13}C NMR spectrum for compound S2

Supplementary References

Supporting synthetic methods

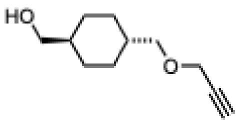

***trans*-{4-[(Prop-2-ynyloxy)methyl]cyclohexyl}methanol (compound S1).**

trans-1,4-Cylohexanediol (13.86 mmol, 2.00g) was dissolved in 28 mL of dry THF. Potassium tert-butoxide (1M solution in THF, 13.86 mmol, 13.86 mL) was then added followed by propargyl bromide (13.86 mmol, 1.34 mL). After stirring at rt for 19 h the mixture was diluted with 50mL of H_2O and 50mL of brine. This mixture was then extracted with ethyl acetate (3 x 50 mL) and the combined extracts were dried ($MgSO_4$) and concentrated. Purification by silica gel chromatography (20-40% ethyl acetate / hexanes) gave 0.401g (16% yield) of *trans*-{4-[(prop-2-ynyloxy)methyl]cyclohexyl}methanol as a yellow oil. This compound has been previously reported [1].

TLC Rf = 0.22 (20% ethyl acetate / hexanes); IR (NaCl) 3431, 3297, 2919, 2853, 2116 cm^{-1}; ^{1}H NMR (400 MHz, $CDCl_3$) d 4.05 (s, 2H), 3.38 (d, *J* = 6.4 Hz, 2H), 3.27 (d, J = 6.4 Hz, 2H), 2.34 (t, J = 2.4 Hz, 1H), 1.81-1.74 (m, 4H), 1.54-1.45 (m, 1H), 1.42-1.33 (m, 1H), 0.97-0.83 (m, 4H); ^{13}C NMR (133 MHz, $CDCl_3$) d 80.0, 75.9, 74.0, 68.6, 58.2, 40.5, 38.1, 29.3, 28.9.

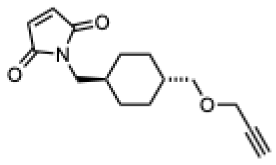

***trans*-1-({4-[(Prop-2-ynyloxy)methyl]cyclohexyl}methyl)-3-pyrroline-2,5-dione (compound S2).**

trans-{4-[(Prop-2-ynyloxy)methyl]cyclohexyl}methanol (**compound S1**, 1.37 mmol, 0.250g) was dissolved in 10 mL of dry THF. Maleimide (1.51 mmol, 0.147g) and triphenylphosphine (1.98 mmol, 0.388 g) was then added followed by DIAD (1.66 mmol, 0.32 mL). The reaction was stirred at room temperature for 18 h and then concentrated. The residue was purified by silica gel chromatography (40-50% ethyl acetate / hexanes) gave 0.071g (21% yield) of *trans*-1-({4-[(prop-2-ynyloxy)methyl]cyclohexyl}methyl)-3-pyrroline-2,5-dione as an off-white powder.

TLC Rf = 0.33 (40% ethyl acetate / hexanes); IR (NaCl) 3271, 3090, 2923, 2852, 2111, 1697 cm^{-1}; ^{1}H NMR (400 MHz, $CDCl_3$) d 6.62 (s, 2H), 4.04 (d, *J* = 2.4 Hz, 2H), 3.29 (d, *J* = 6.8 Hz, 2H), 3.23 (d, *J* = 6.5 Hz, 2H), 2.33 (t, *J* = 2.4 Hz, 1H), 1.76-1.72 (m, 2H), 1.62-1.59 (m, 2H), 1.48-1.46 (m, 1H), 1.25-1.13 (m, 1H), 0.96-0.79 (m, 4H); ^{13}C NMR (133 MHz, $CDCl_3$) d 171.0, 134.0, 80.0, 75.7, 74.1, 58.2, 43.9, 37.7, 37.1, 30.2, 29.1.

Table S1. Analytical information for peptide ligands. The letters "a" or "b" indicated after each PEG3 linker reference which linker was used each construct. Structures of the different PEG3 linkers are provided in Figure 7. Purity was determined by HPLC peak integration of the purified peptide.

Ligand	Sequence	$[M+H]^+$ predicted	$[M+H]^+$ found (MALDI)	Purity (%)	Retention Time (min)
3	H-GS-Dap-Nal-1-LSPT-Mini-PEG3a-C-NH_2	1149.55	1149.261	95	13.4
3'	H-GS-Dap-Nal-1-LSPT-Mini-PEG3b-C-NH_2	1134.54	1134.343	97	12.8
4	H-GS-Dap-Nal-1-LSPT-Mini-PEG3a-AHA-NH_2	1172.60	1172.321	97	13.5
5	H-GS-Dap-Nal-1-LSPT-Mini-PEG3a-C-Linker	1410.69	1410.372	96	16.7
6	H-GS-Dap-Nal-1-LSPT-Mini-PEG3a-AHA-Linker	1433.79	1433.498	95	15.5
7	H-GS-Dap-Nal-1-LSPT-Mini-PEG3a-C-Linker-AF488azide	2027.77	2027.292	99	15.5
8	H-GS-Dap-Nal-1-LSPT-Mini-PEG3-C-Linker-FAM5azide	1868.81	1868.683	96	16.7
9	H-GS-Dap-Nal-1-LSPT-Mini-PEG3a-C-Linker-SulfoCy5azide	2135.97	2135.896	>99	15.5
10	H-GS-Dap-Nal-1-LSPT-Mini-PEG3a-C-Linker-TAMRAazide	2040.97	2040.135	99	16.5
11	H-GS-Dap-Nal-1-LSPT-Mini-PEG3a-C-Linker-TideQuencher2azide	1860.25	1860.648	95	19.5
12	H-GS-Dap-Nal-1-LSPT-Mini-PEG3-C-Linker-AzoDye1azide	1983.97	1983.016	>99	22.7
13	H-GS-Dap-Nal-1-LSPT-Mini-PEG3a-(Biotin-K)-NH_2	1400.72	1400.054	>99	13.4
13'	H-GS-Dap-Nal-1-LSPT-Mini-PEG3b-(Biotin-K)-NH_2	1386.70	1386.562	94	13.4
14	H-GS-Dap-Nal-1-LSPT-Mini-PEG3a-AHA-Linker-Mertansine	2170.01	2170.241	99	19.3
15	H-GS-Dap-Nal-1-LSPT-Mini-PEG3a-AHA-AF488alkyne	1742.63	1742.360	96	13.1
16	H-GS-Dap-Nal-1-LSPT-Mini-PEG3a-C-SulfoCy5maleimide	1914.20	1914.417	>99	14.3
16'	H-GS-Dap-Nal-1-LSPT-Mini-PEG3b-C-SulfoCy5maleimide	1900.20	1900.968	93	14.4
17	H-GS-Dap-Nal-1-LSPT-Mini-PEG3a-AHA-AzoDye1alkyne	1713.92	1713.714	99	13.2

Table S2. Ligand-conjugate GOAT inhibitory activity. The letters "a" or "b" indicated after each PEG3 linker reference which linker was used each construct. Structures of the different PEG3 linkers are provided in Figure 7. All reported IC_{50} values against hGOAT represent the average of three independent trials.

Ligand	Sequence	Linker (PEG3 a or b)	+/- Bifunctional Adapter	Cargo Type	IC_{50} (nM)
3	H-GS-Dap-Nal-1-LSPT-Mini-PEG3a-C-NH_2	-	-	-	28.9 ± 8.7
3'	H-GS-Dap-Nal-1-LSPT-Mini-PEG3b-C-NH_2	-	-	Fluorophore (SulfoCy5)	110.4 ± 13.5
4	H-GS-Dap-Nal-1-LSPT-Mini-PEG3a-AHA-NH_2	a	-	-	31 ± 15.8
5	H-GS-Dap-Nal-1-LSPT-Mini-PEG3a-C-Linker	b	-	-	13 ± 1
6	H-GS-Dap-Nal-1-LSPT-Mini-PEG3a-AHA-Linker	a	-	-	1053.6 ± 79.5
7	H-GS-Dap-Nal-1-LSPT-Mini-PEG3a-C-Linker-AF488azide	a	+	-	102.8 ± 14
8	H-GS-Dap-Nal-1-LSPT-Mini-PEG3-C-Linker-FAM5azide	a	+	-	73.8 ± 4.8
9	H-GS-Dap-Nal-1-LSPT-Mini-PEG3a-C-Linker-SulfoCy5azide	a	+	Fluorophore (AF488 azide)	84.4 ± 11.5
10	H-GS-Dap-Nal-1-LSPT-Mini-PEG3a-C-Linker-TAMRAazide	a	+	Fluorophore (FAM5 azide)	16.4 ± 1.6
11	H-GS-Dap-Nal-1-LSPT-Mini-PEG3a-C-Linker-TideQuencher2azide	a	+	Fluorophore (SulfoCy5 azide)	25.7 ± 6.2
12	H-GS-Dap-Nal-1-LSPT-Mini-PEG3-C-Linker-AzoDye1azide	a	+	Fluorophore (TAMRA)	5.9 ± 1.7
13	H-GS-Dap-Nal-1-LSPT-Mini-PEG3a-(Biotin-K)-NH_2	a	+	Quencher (TideQuencher2 azide)	51.8 ± 5.7
13'	H-GS-Dap-Nal-1-LSPT-Mini-PEG3b-(Biotin-K)-NH_2	a	+	Quencher (AzoDye1 azide)	610.9 ± 70.9
14	H-GS-Dap-Nal-1-LSPT-Mini-PEG3a-AHA-Linker-Mertansine	a	-	Affinity (Biotin)	18.8 ± 5
15	H-GS-Dap-Nal-1-LSPT-Mini-PEG3a-AHA-AF488alkyne	b	-	Affinity (Biotin)	39.1 ± 8.5
16	H-GS-Dap-Nal-1-LSPT-Mini-PEG3a-C-SulfoCy5maleimide	a	+	Cytotoxin (Mertansine)	56.8 ± 8.6
16'	H-GS-Dap-Nal-1-LSPT-Mini-PEG3b-C-SulfoCy5maleimide	a	-	Fluorophore (AF488 alkyne)	33.1 ± 7.4
17	H-GS-Dap-Nal-1-LSPT-Mini-PEG3a-AHA-AzoDye1alkyne	a	-	Fluorophore (SulfoCy5)	3.9 ± 0.4

A)

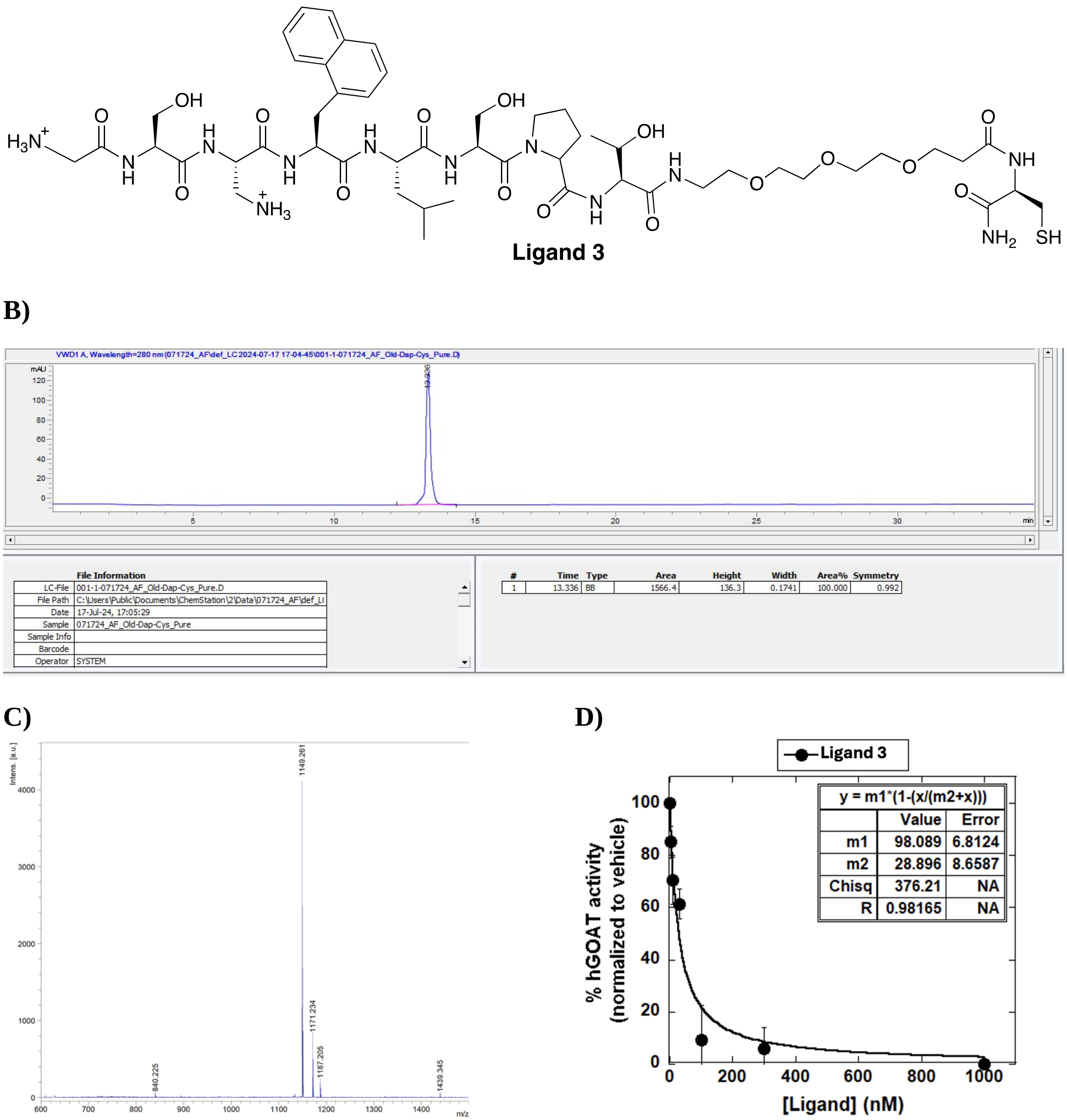

Figure S1. Characterization information for Ligand 3. A) Chemical structure of Ligand **3**. B) RP-HPLC chromatogram of Ligand **3** with retention time at 13.3 minutes depicting purity. C) MALDI-TOF spectrum of purified Ligand **3** with $[M+H]^+$ = 1149.261. D) IC_{50} value for Ligand **3** against hGOAT (IC_{50}= 28.9 ± 8.7 nM). IC_{50} values against hGOAT represent the average of three independent trials. All protocols are reported in the Methods section.

A)

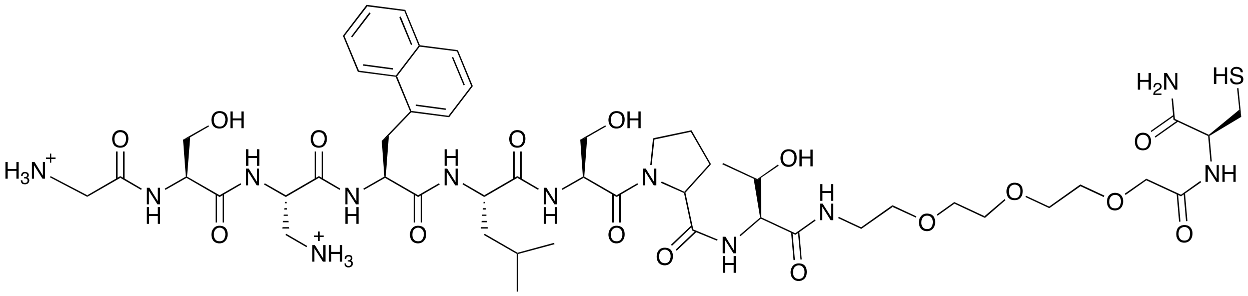

Ligand 3’

B)

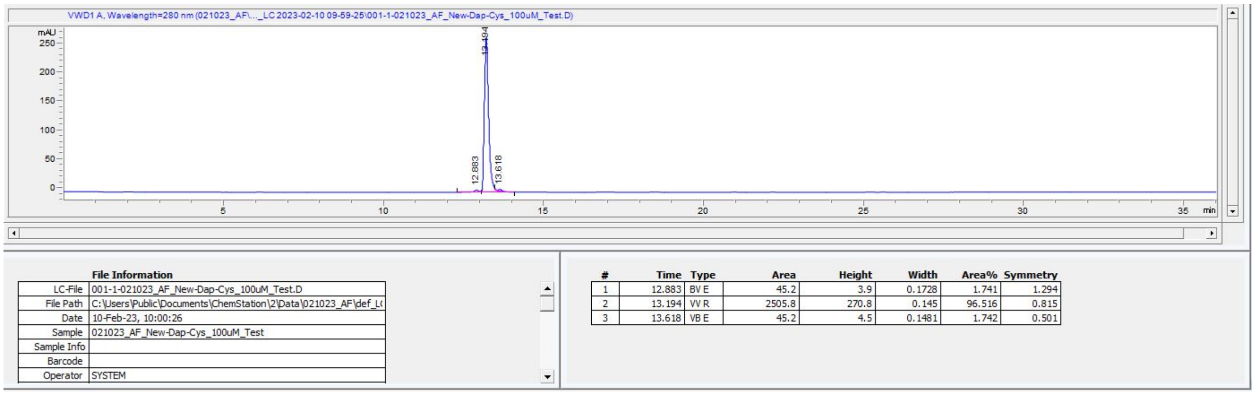

C)

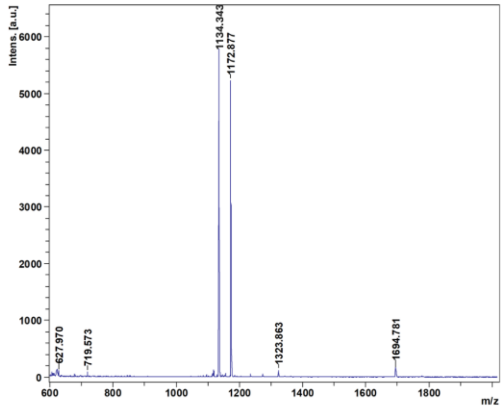

D)

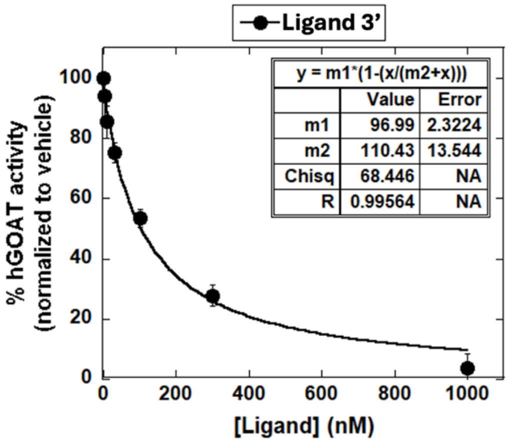

Figure S2. Characterization information for Ligand 3’. A) Chemical structure of Ligand **3’**. B) RP-HPLC chromatogram of Ligand **3’** with retention time at 13.2 minutes depicting purity. C) MALDI-TOF spectrum of purified Ligand **3’** with $[M+H]^+$ = 1134.434. D) IC_{50} value for Ligand **3’** against hGOAT (IC_{50}= 110.4 ± 13.5 nM). IC_{50} values against hGOAT represent the average of three independent trials. All protocols are reported in the Methods section.

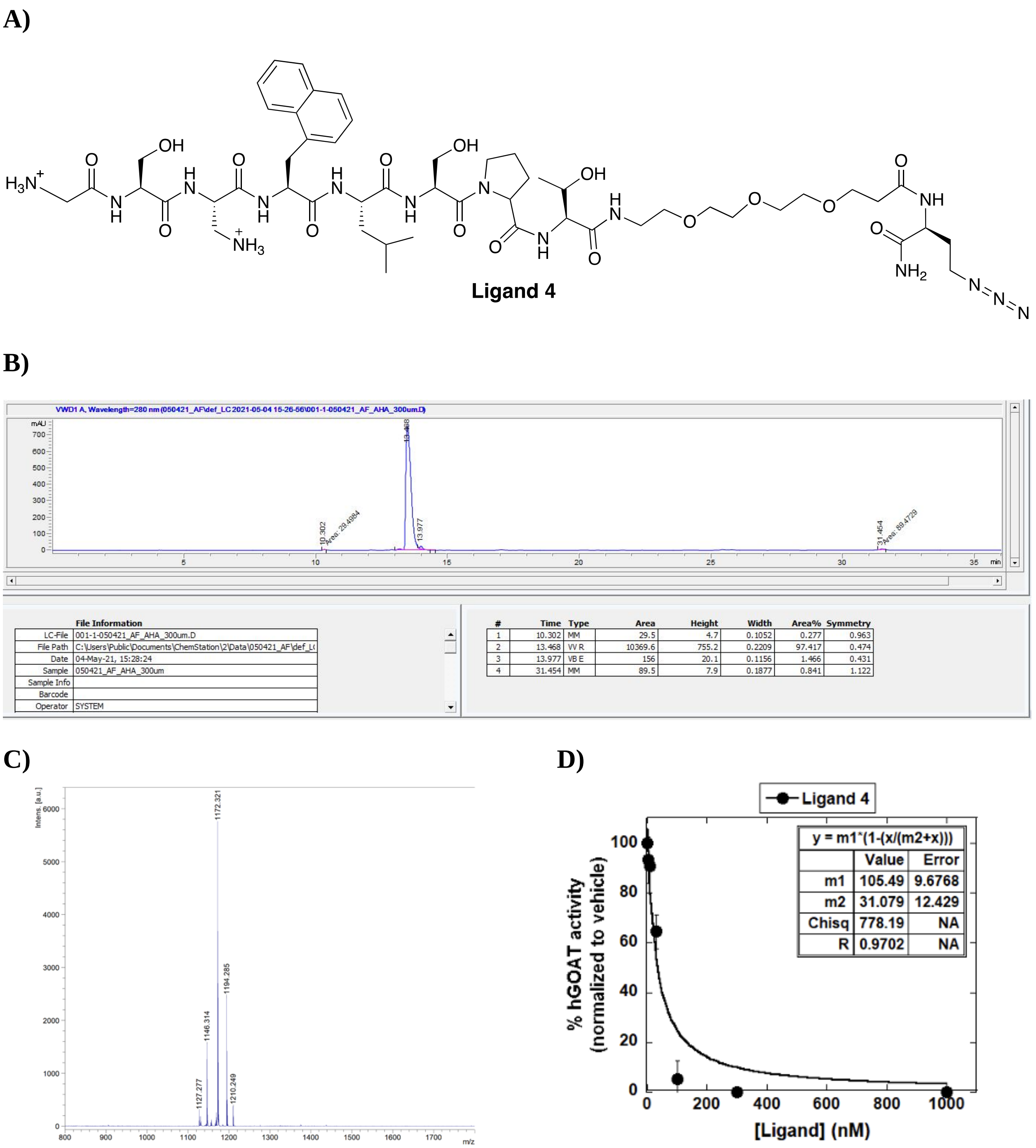

Figure S3. Characterization information for Ligand 4. A) Chemical structure of Ligand **4**. B) RP-HPLC chromatogram of Ligand **4** with retention time at 13.2 minutes depicting purity. C) MALDI-TOF spectrum of purified Ligand **4** with $[M+H]^+$ =1172.321. D) IC_{50} value for Ligand **4** against hGOAT (IC_{50}= 31.0 ± 12.4 nM). IC_{50} values against hGOAT represent the average of three independent trials. All protocols are reported in the Methods section.

A)

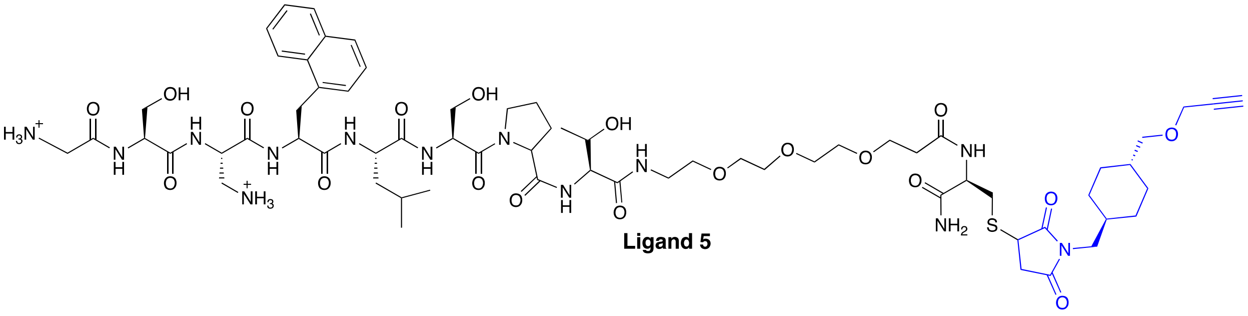

B)

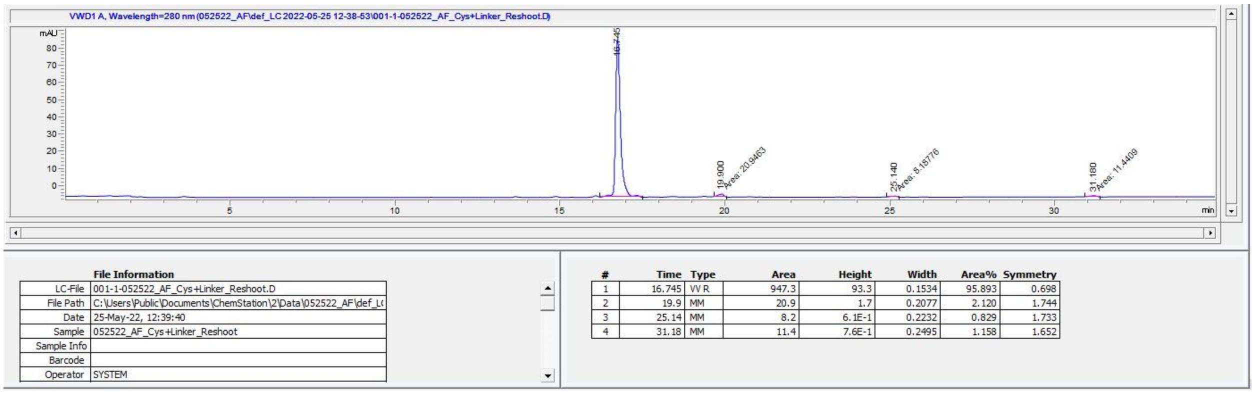

C)

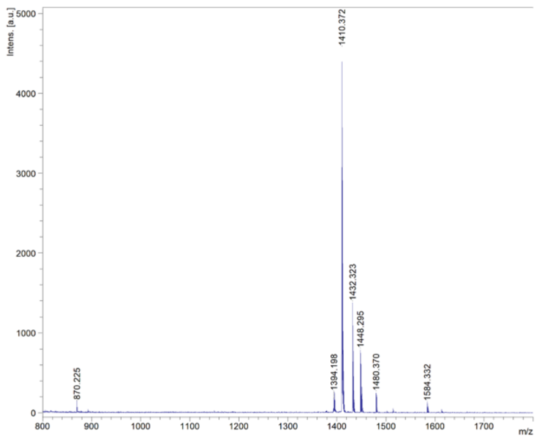

D)

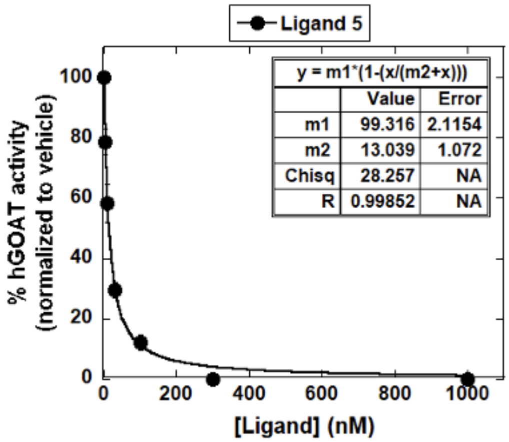

Figure S4. Characterization information for Ligand 5. A) Chemical structure of Ligand **5**. B) RP-HPLC chromatogram of Ligand **5** with retention time at 16.7 minutes depicting purity. C) MALDI-TOF spectrum of purified Ligand **5** with $[M+H]^+$ = 1410.372. D) IC_{50} value for Ligand **5** against hGOAT (IC_{50}= 13.0 ± 1.0 nM). IC_{50} values against hGOAT represent the average of three independent trials. All protocols are reported in the Methods section.

A)

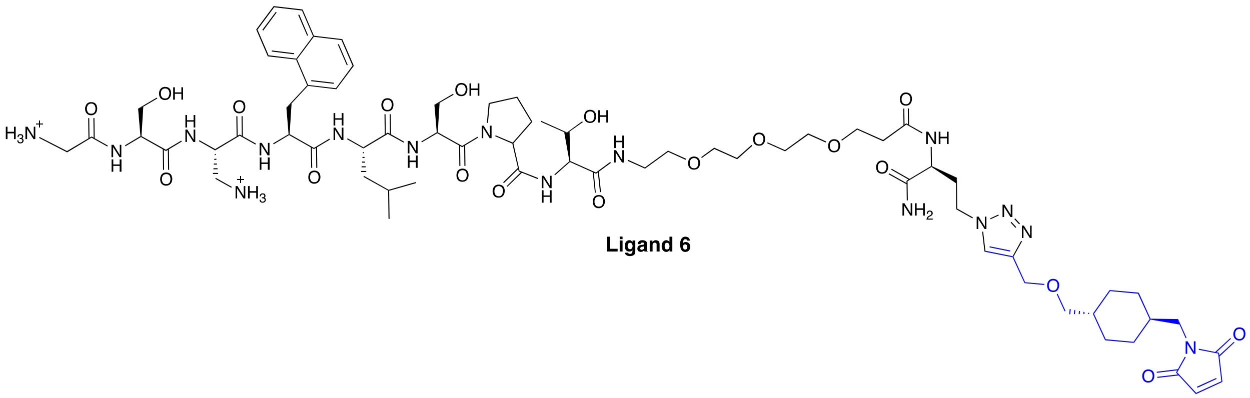

B)

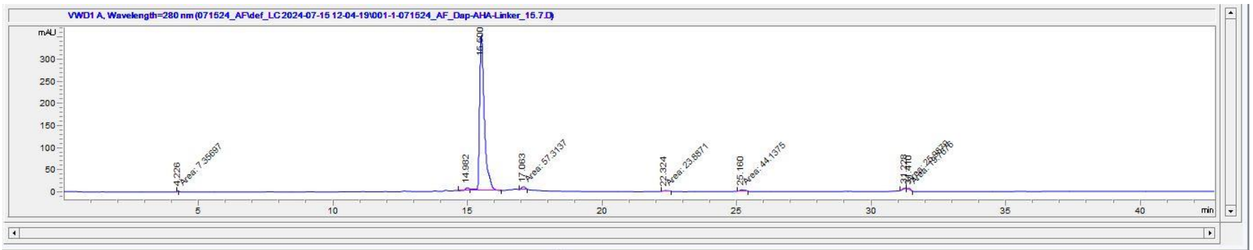

File Information

LC-File	001-1-071524_AF_Dap-AHA-Linker_15.7.D
File Path	C:\Users\Public\Documents\ChemStation\2\Data\071524_AF\def_L(
Date	15-Jul-24, 12:04:57
Sample	071524_AF_Dap-AHA-Linker_15.7
Sample Info	
Barcode	
Operator	SYSTEM

#	Time	Type	Area	Height	Width	Area%	Symmetry
1	4.226	MM	7.4	3.3	0.0367	0.162	0.965
2	14.982	BV E	67.2	6.4	0.1581	1.484	1.073
3	15.5	VB R	4285.3	350.3	0.1852	94.577	0.602
4	17.063	MM	57.3	6	0.1589	1.265	0.937
5	22.324	MM	23.9	1.8	0.2245	0.527	0.353
6	25.16	MM	44.1	3	0.2438	0.974	0.391
7	31.228	MM	26	3.2	0.1356	0.574	1.776

C)

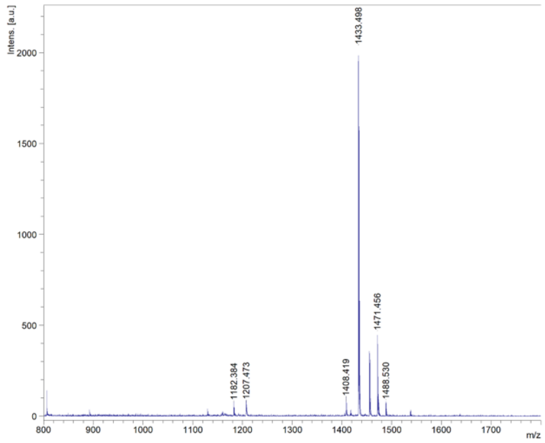

D)

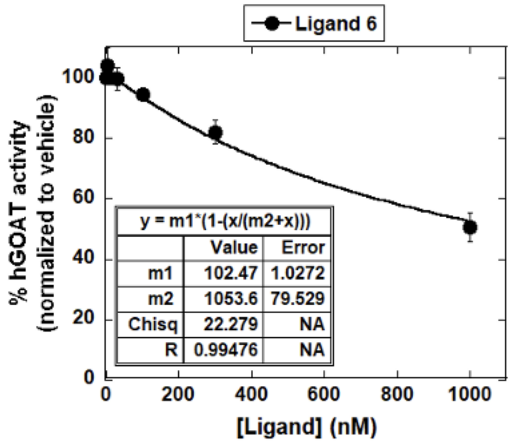

Figure S5. Characterization information for Ligand 6. A) Chemical structure of Ligand **6**. B) RP-HPLC chromatogram of Ligand **6** with retention time at 15.5 minutes depicting purity. C) MALDI-TOF spectrum of purified Ligand **6** with $[M+H]^+ = 1433.498$. D) IC_{50} value for Ligand **6** against hGOAT ($IC_{50} = 1053.6 \pm 79.5$ nM). IC_{50} values against hGOAT represent the average of three independent trials. All protocols are reported in the Methods section.

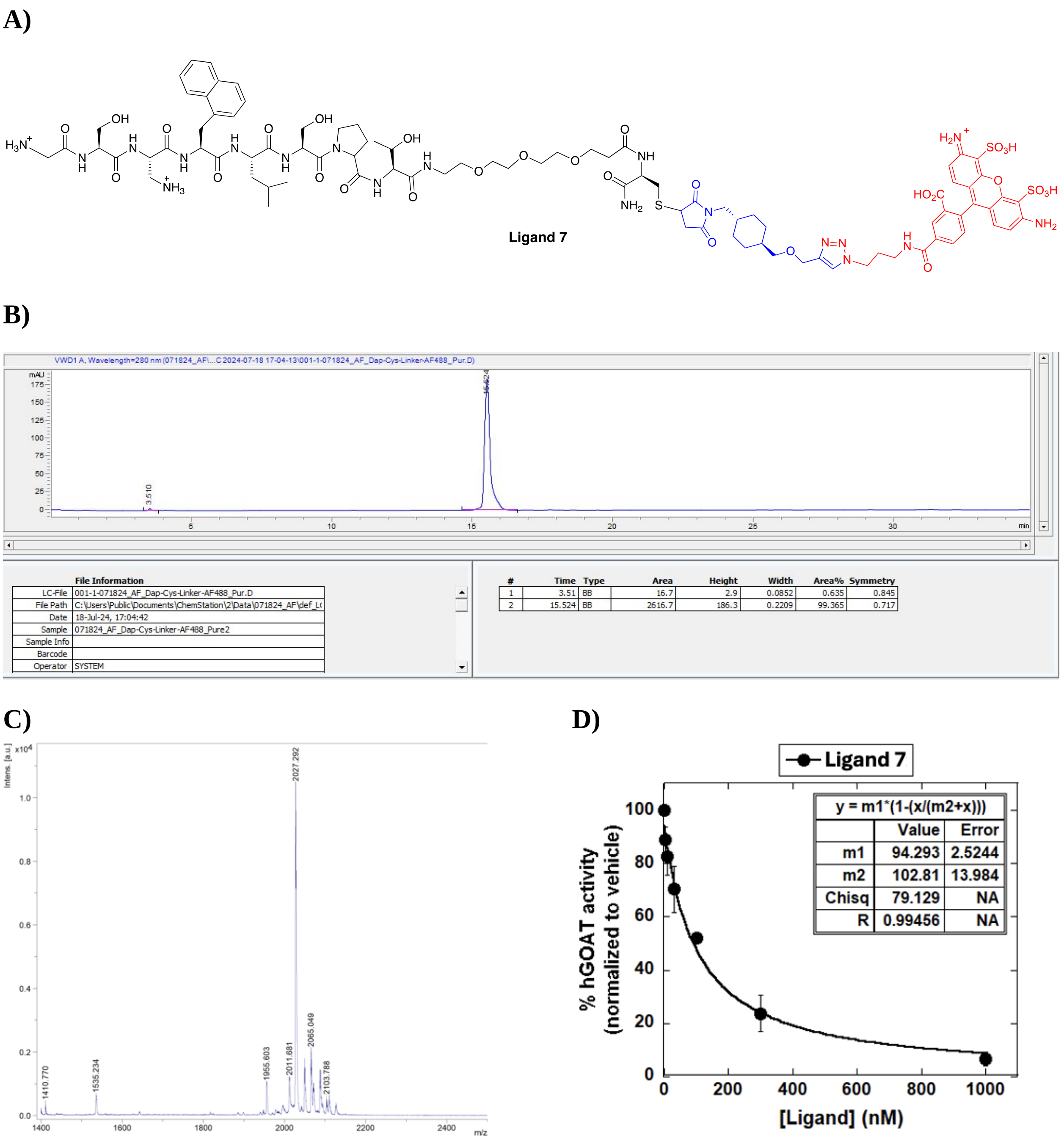

Figure S6. Characterization information for Ligand 7. A) Chemical structure of Ligand **7**. B) RP-HPLC chromatogram of Ligand **7** with retention time at 15.5 minutes depicting purity. C) MALDI-TOF spectrum of purified Ligand **7** with $[M+H]^+$ = 2027.292. D) IC_{50} value for Ligand **7** against hGOAT (IC_{50}= 102.8 ± 14.0 nM). IC_{50} values against hGOAT represent the average of three independent trials. All protocols are reported in the Methods section.

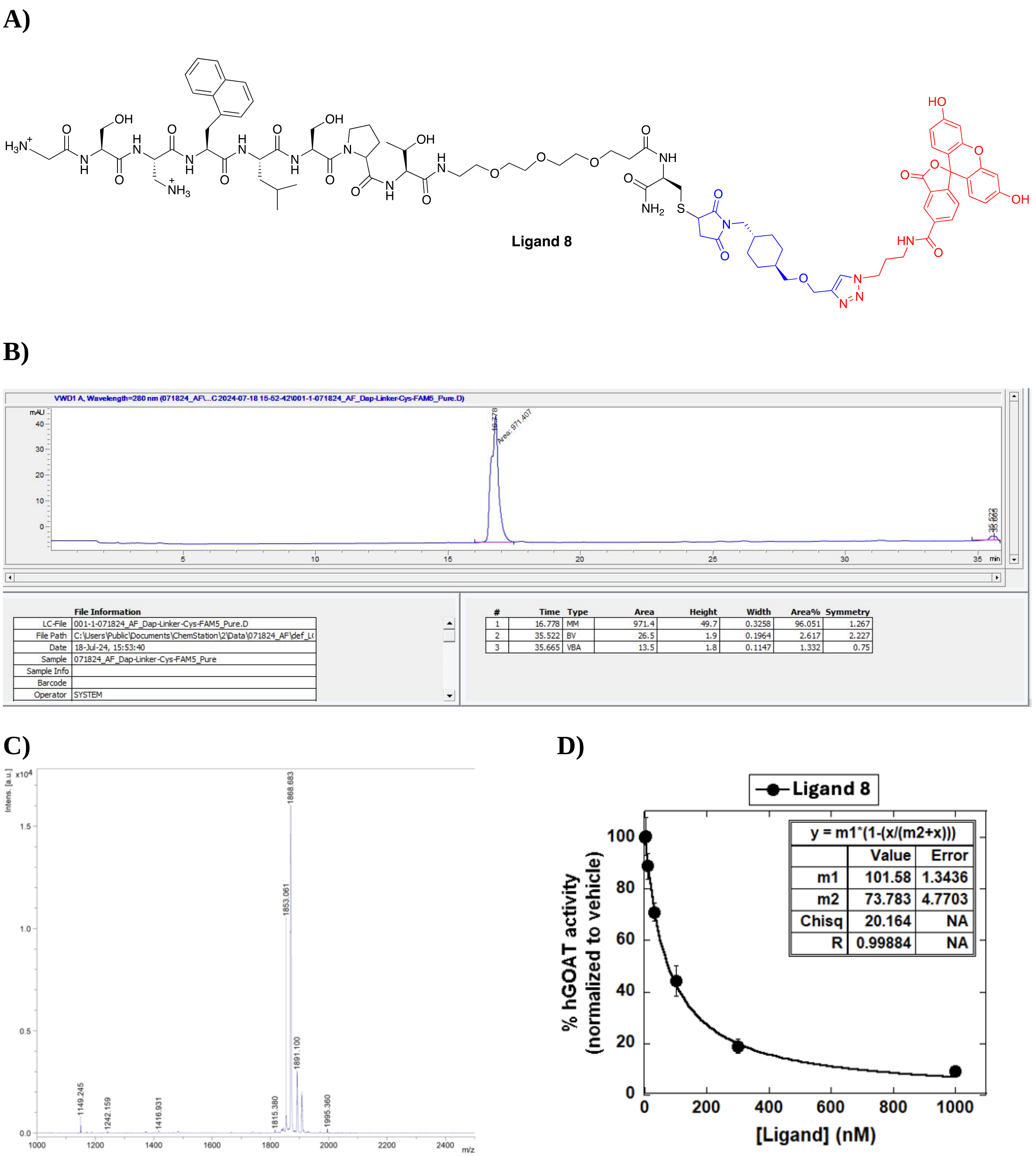

Figure S7. Characterization information for Ligand 8. A) Chemical structure of Ligand **8**. B) RP-HPLC chromatogram of Ligand **8** with retention time at 16.7 minutes depicting purity. C) MALDI-TOF spectrum of purified Ligand **8** with $[M+H]^+$ = 1868.683. D) IC_{50} value for Ligand **8** against hGOAT (IC_{50}= 73.8 ± 4.8 nM). IC_{50} values against hGOAT represent the average of three independent trials. All protocols are reported in the Methods section.

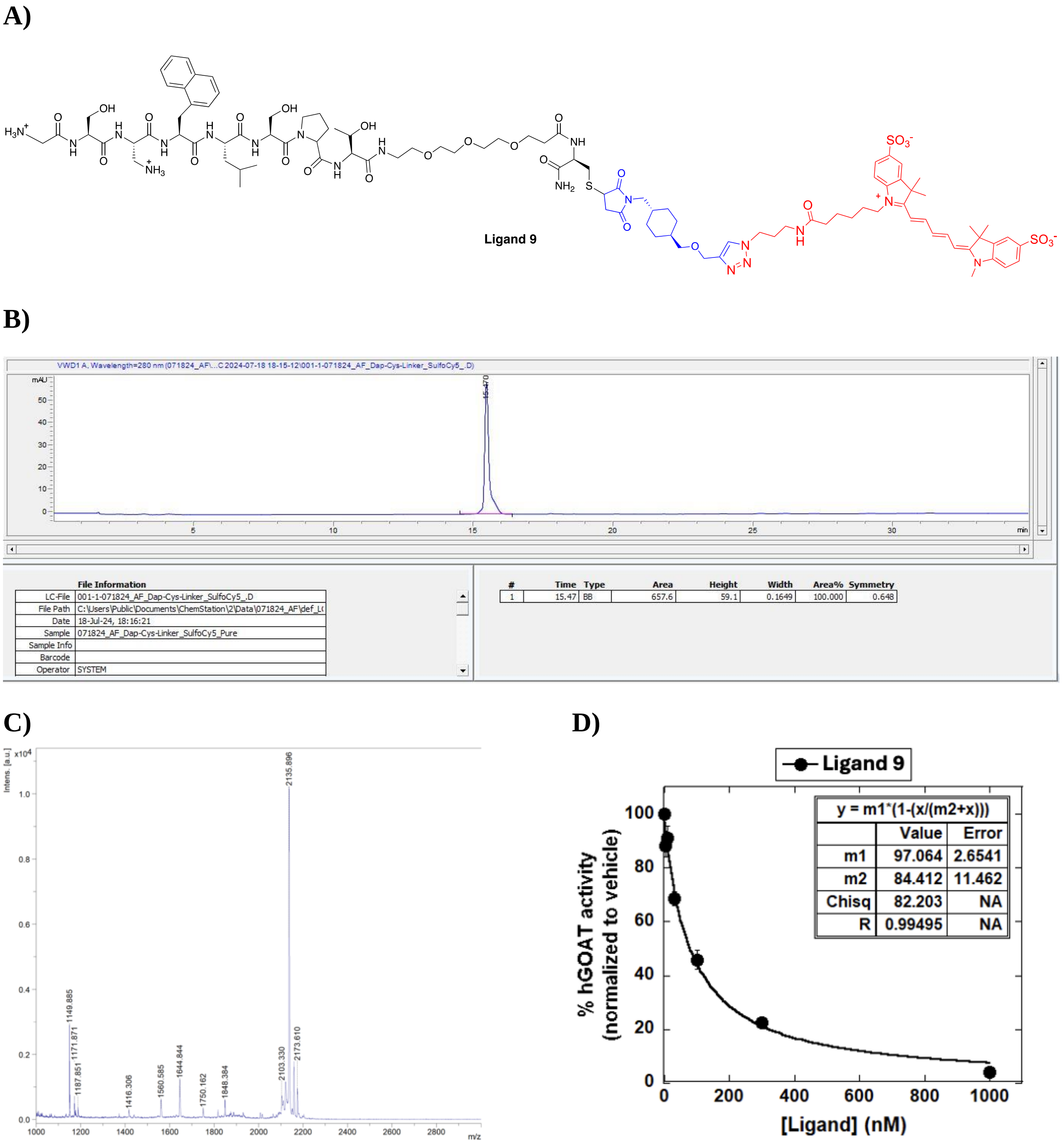

Figure S8. Characterization information for Ligand 9. A) Chemical structure of Ligand **9**. B) RP-HPLC chromatogram of Ligand **9** with retention time at 15.5 minutes depicting purity. C) MALDI-TOF spectrum of purified Ligand **9** with $[M+H]^+$ = 2135.896. D) IC_{50} value for Ligand **9** against hGOAT (IC_{50}= 84.4 ± 11.5 nM). IC_{50} values against hGOAT represent the average of three independent trials. All protocols are reported in the Methods section.

A)

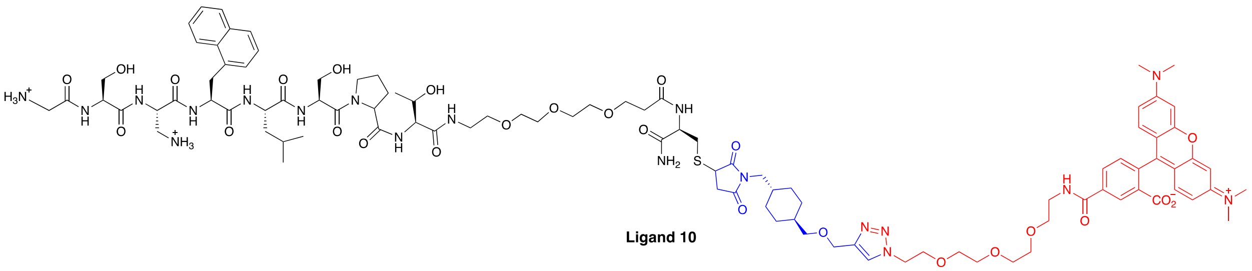

B)

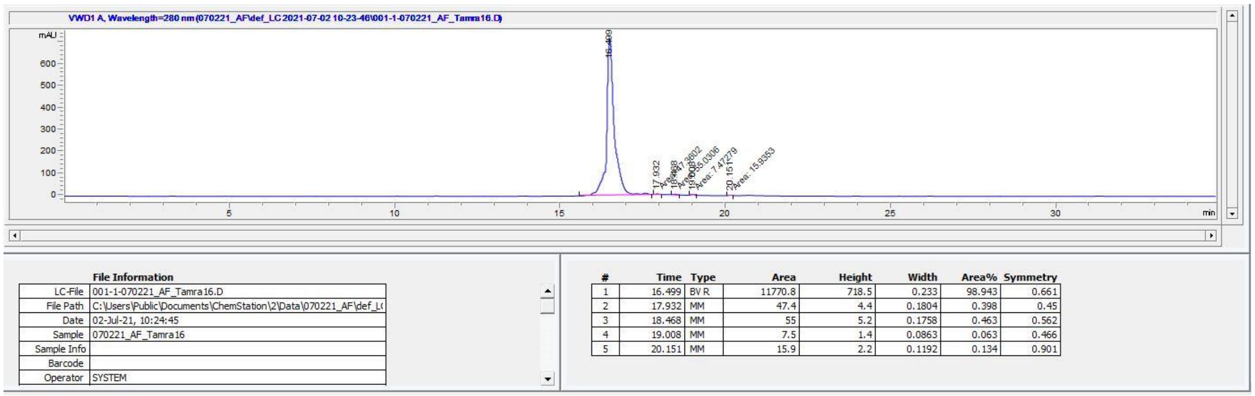

C)

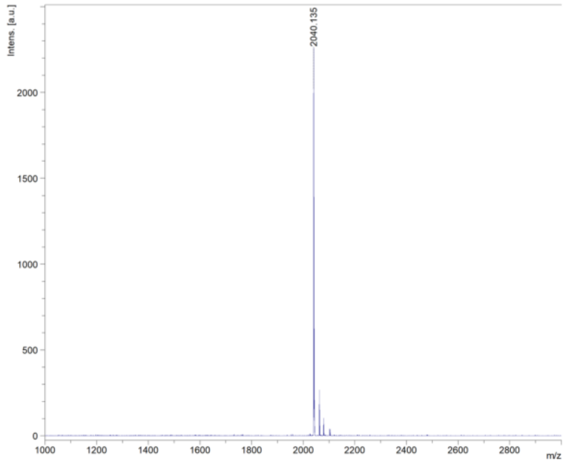

D)

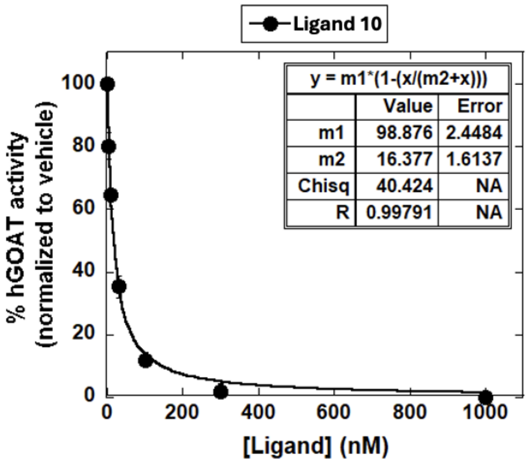

Figure S9. Characterization information for Ligand 10. A) Chemical structure of Ligand **10**. B) RP-HPLC chromatogram of Ligand **10** with retention time at 16.4 minutes depicting purity. C) MALDI-TOF spectrum of purified Ligand **10** with $[M+H]^+$ = 2040.135. D) IC_{50} value for Ligand **10** against hGOAT (IC_{50}= 16.4 ± 1.6 nM). IC_{50} values against hGOAT represent the average of three independent trials. All protocols are reported in the Methods section.

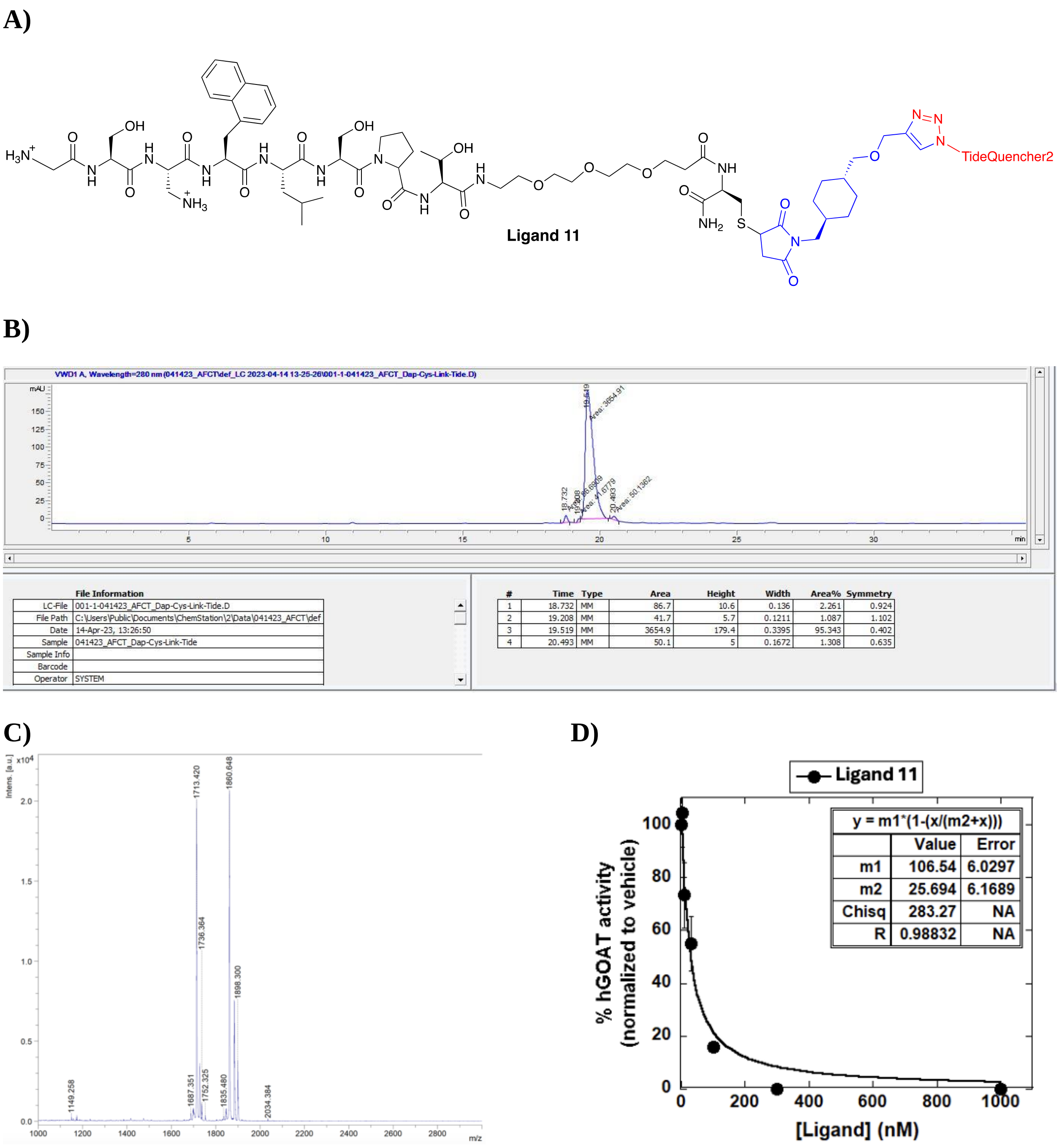

Figure S10. Characterization information for Ligand 11. A) Chemical structure of Ligand **11**. B) RP-HPLC chromatogram of Ligand **11** with retention time at 19.5 minutes depicting purity. C) MALDI-TOF spectrum of purified Ligand **11** with $[M+H]^+$ = 1860.648. D) IC_{50} value for Ligand **11** against hGOAT (IC_{50}= 25.7 ± 6.2 nM). IC_{50} values against hGOAT represent the average of three independent trials. All protocols are reported in the Methods section.

A)

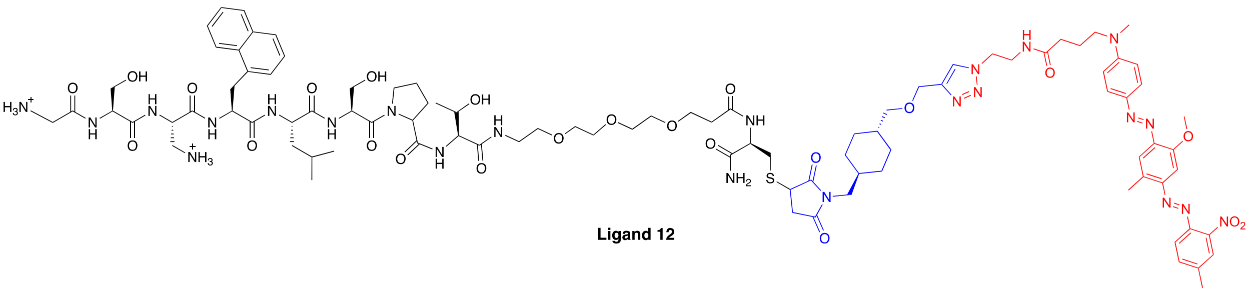

B)

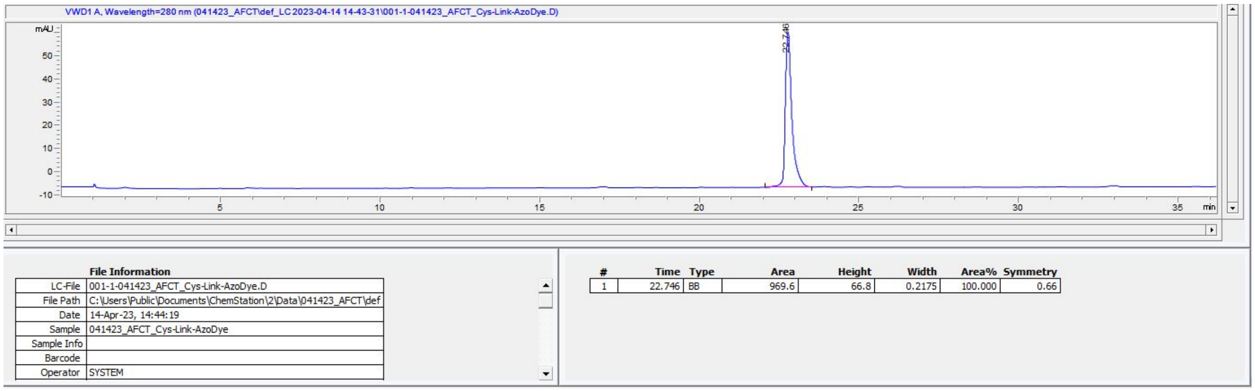

C)

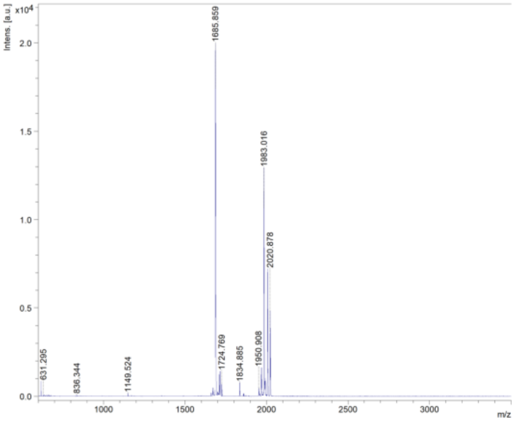

D)

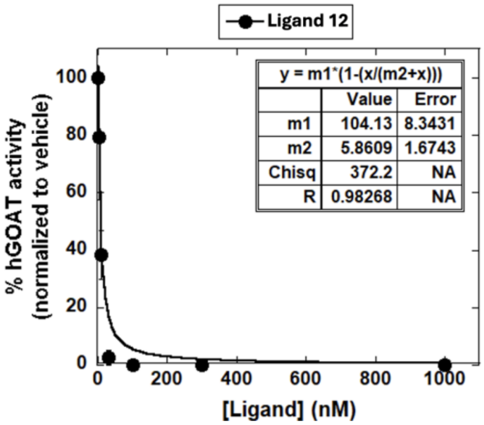

Figure S11. Characterization information for Ligand 12. A) Chemical structure of Ligand **12**. B) RP-HPLC chromatogram of Ligand **12** with retention time at 22.7 minutes depicting purity. C) MALDI-TOF spectrum of purified Ligand **12** with $[M+H]^+$ = 1983.016. D) IC_{50} value for Ligand **12** against hGOAT (IC_{50}= 5.9 ± 1.7 nM. IC_{50} values against hGOAT represent the average of three independent trials. All protocols are reported in the Methods section.

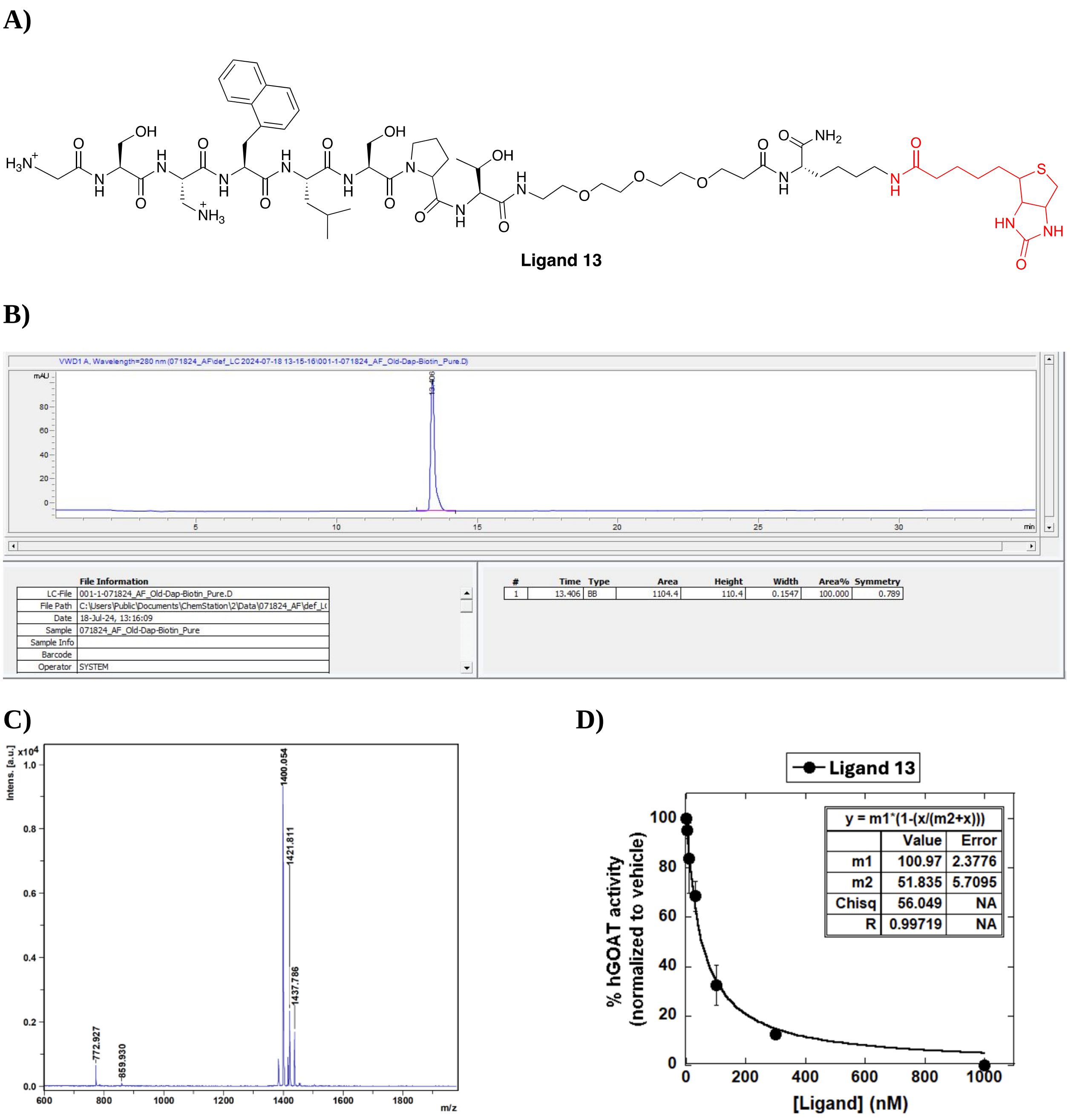

Figure S12. Characterization information for Ligand 13. A) Chemical structure of Ligand **13**. B) RP-HPLC chromatogram of Ligand **13** with retention time at 13.4 minutes depicting purity. C) MALDI-TOF spectrum of purified Ligand **13** with $[M+H]^+$ = 1400.054. D) IC_{50} value for Ligand **13** against hGOAT (IC_{50}= 51.9 ± 5.7 nM). IC_{50} values against hGOAT represent the average of three independent trials. All protocols are reported in the Methods section.

A)

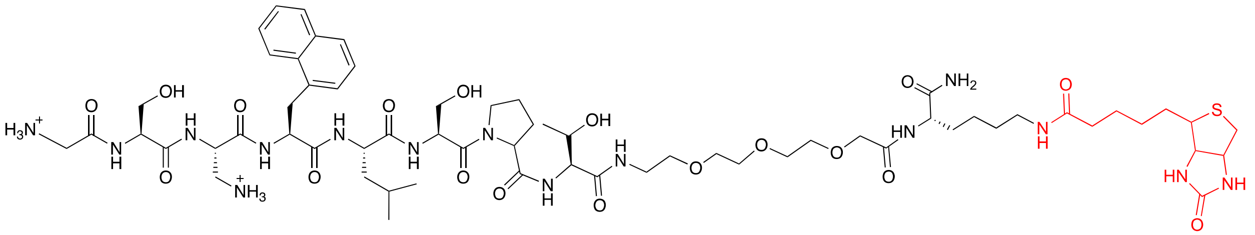

Ligand 13'

B)

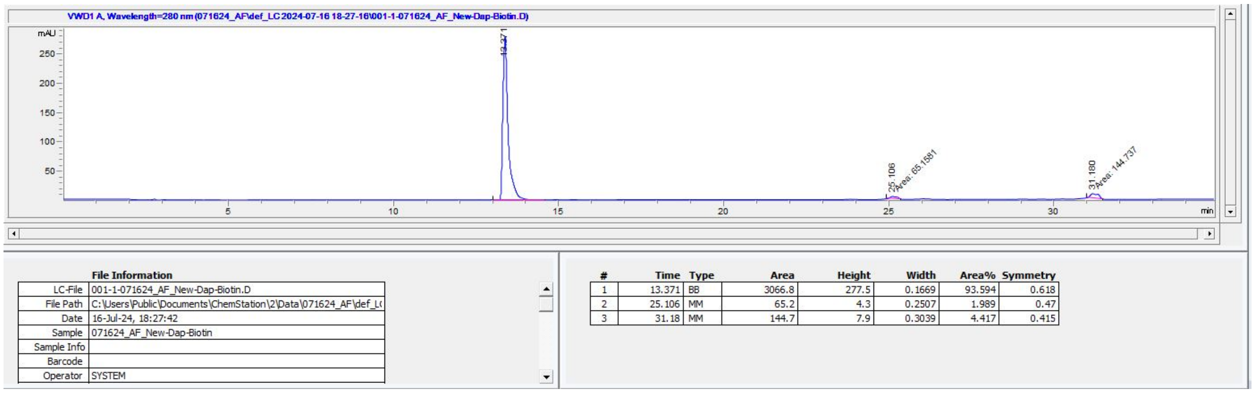

C)

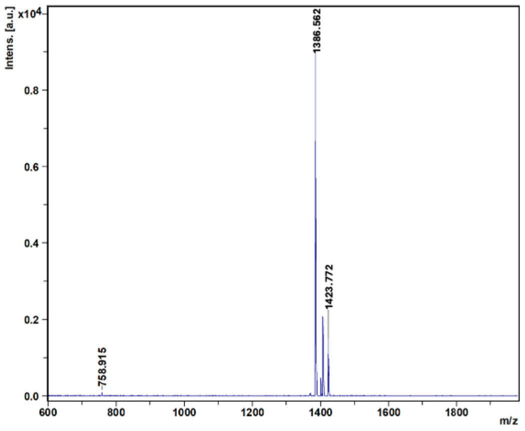

D)

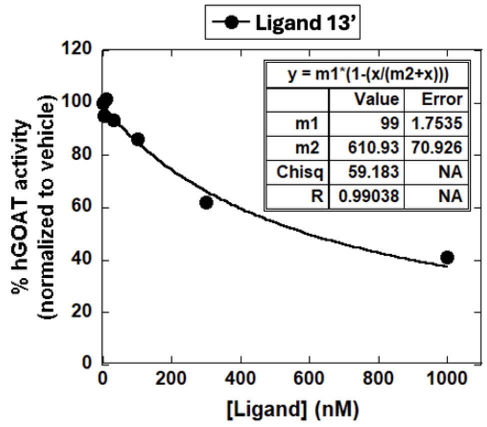

Figure S13. Characterization information for Ligand 13'. A) Chemical structure of Ligand **13'**. B) RP-HPLC chromatogram of Ligand **13'** with retention time at 13.4 minutes depicting purity. C) MALDI-TOF spectrum of purified Ligand **13'** with $[M+H]^+ = 1386.562$. D) IC_{50} value for Ligand **13'** against hGOAT (IC_{50}= 610.9 ± 70.9 nM). IC_{50} values against hGOAT represent the average of three independent trials. All protocols are reported in the Methods section.

A)

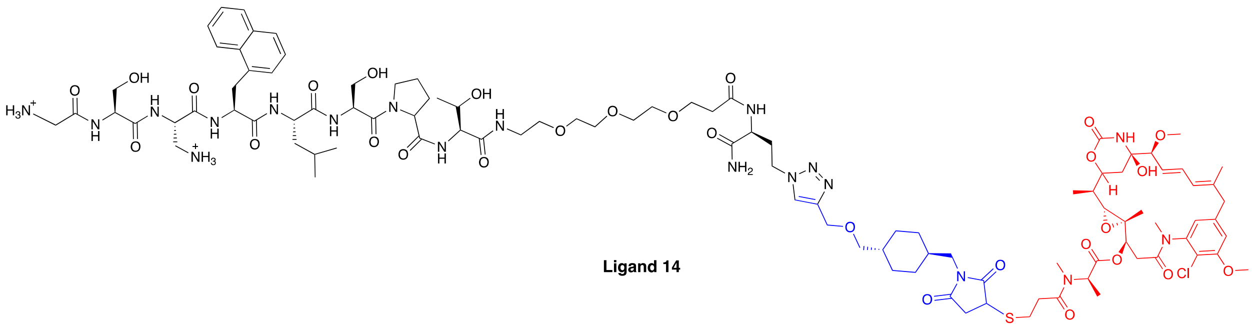

B)

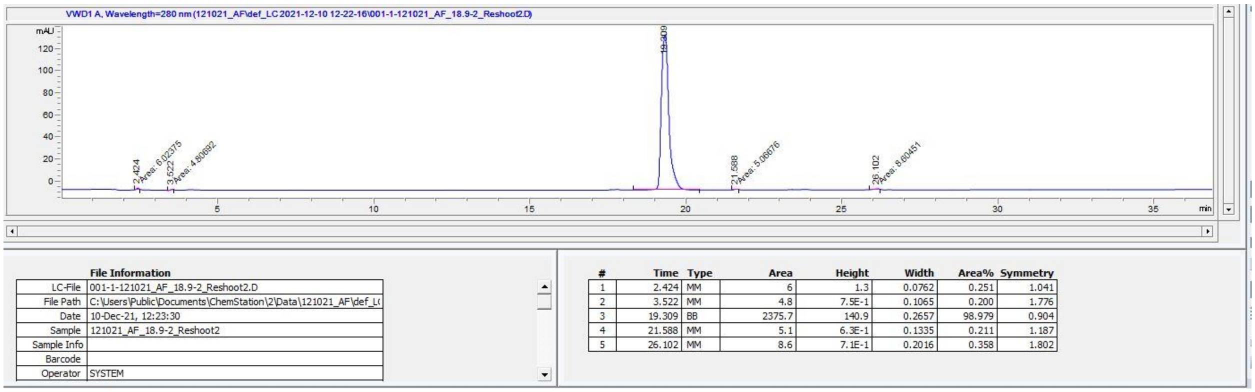

C)

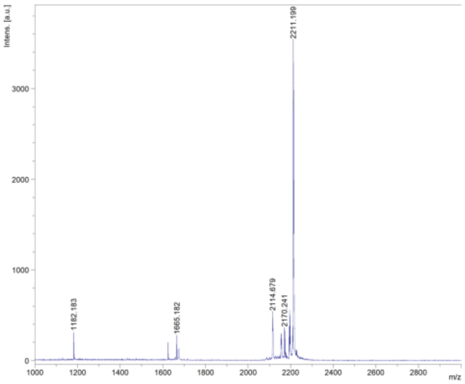

D)

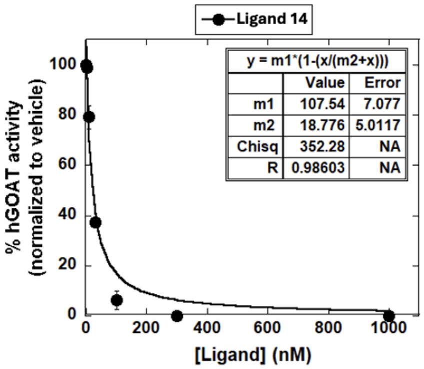

Figure S14. Characterization information for Ligand 14. A) Chemical structure of Ligand **14**. B) RP-HPLC chromatogram of Ligand **14** with retention time at 19.3 minutes depicting purity. C) MALDI-TOF spectrum of purified Ligand **14** with $[M+H]^+$ = 2170.241. D) IC_{50} value for Ligand **14** against hGOAT (IC_{50}= 18.8 ± 5.0 nM). IC_{50} values against hGOAT represent the average of three independent trials. All protocols are reported in the Methods section.

A)

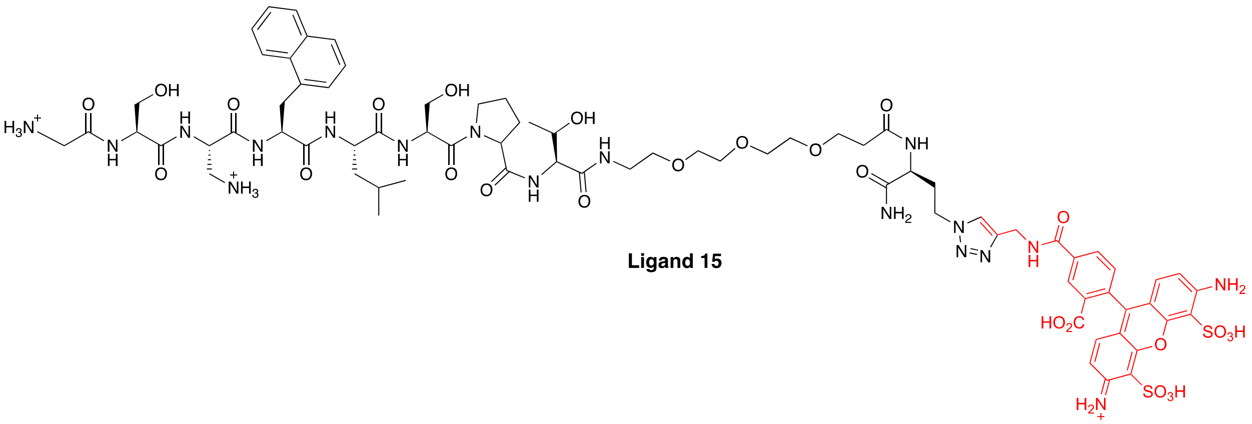

B)

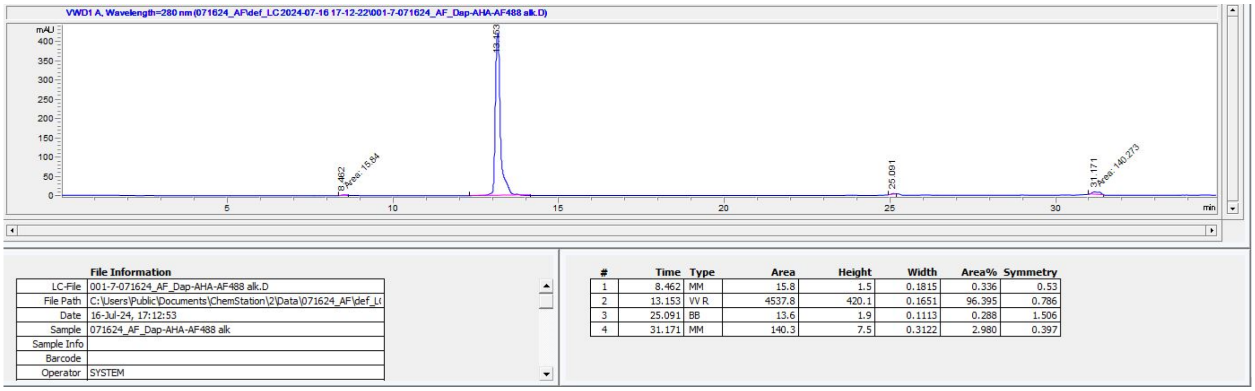

C)

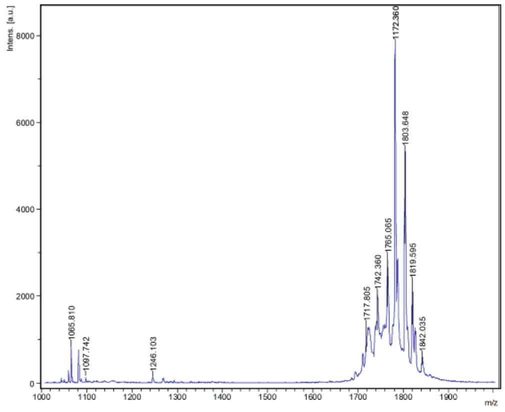

D)

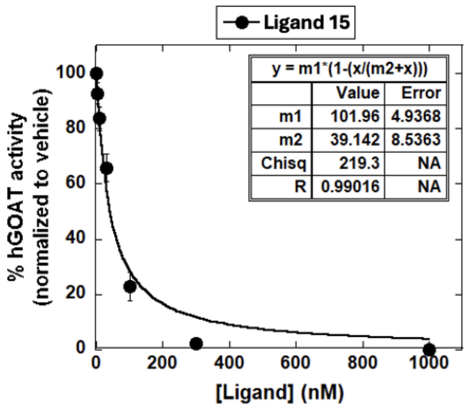

Figure S15. Characterization information for Ligand 15. A) Chemical structure of Ligand **15**. B) RP-HPLC chromatogram of Ligand **15** with retention time at 13.2 minutes depicting purity. C) MALDI-TOF spectrum of purified Ligand **15** with $[M+H]^+ = 1742.360$. D) IC_{50} value for Ligand **15** against hGOAT ($IC_{50} = 39.1 \pm 8.5$ nM). IC_{50} values against hGOAT represent the average of three independent trials. All protocols are reported in the Methods section.

A)

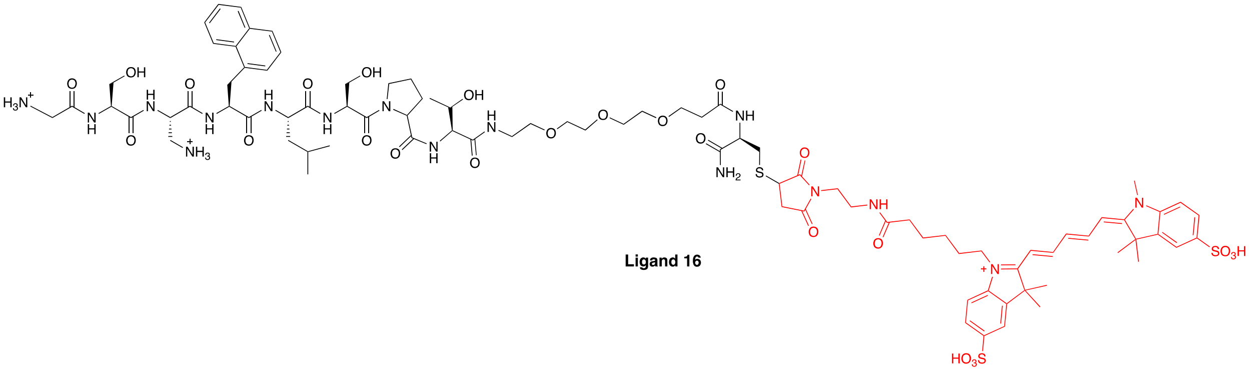

B)

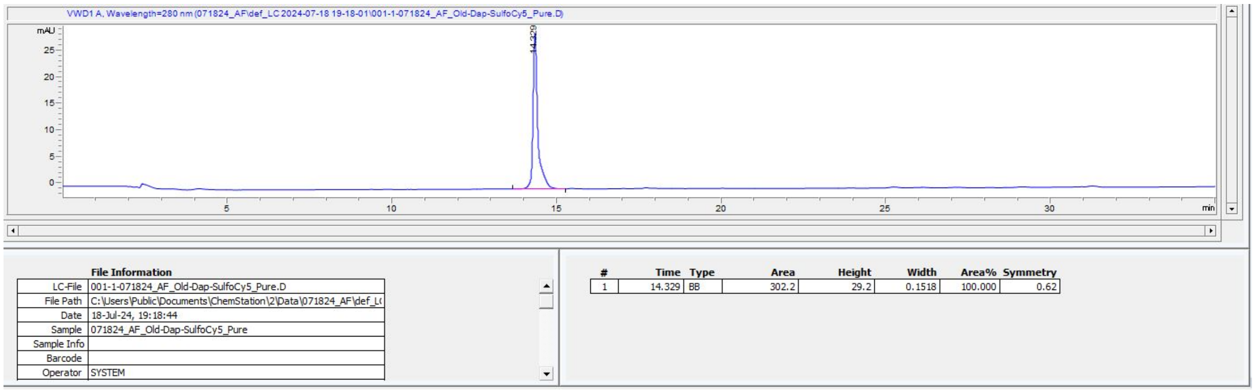

C) **D)**

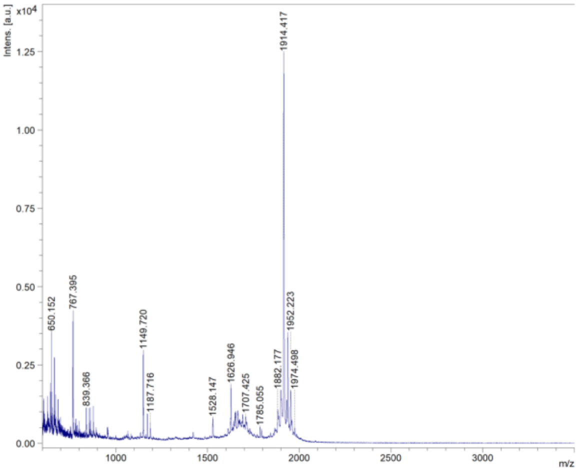

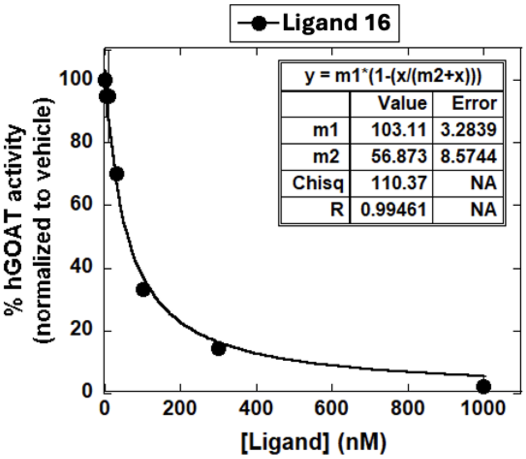

Figure S16. Characterization information for Ligand 16. A) Chemical structure of Ligand **16**. B) RP-HPLC chromatogram of Ligand **16** with retention time at 14.3 minutes depicting purity. C) MALDI-TOF spectrum of purified Ligand **16** with $[M+H]^+ = 1914.417$. D) IC_{50} value for Ligand **16** against hGOAT (IC_{50}= 56.9 ± 8.6 nM). IC_{50} values against hGOAT represent the average of three independent trials. All protocols are reported in the Methods section.

A)

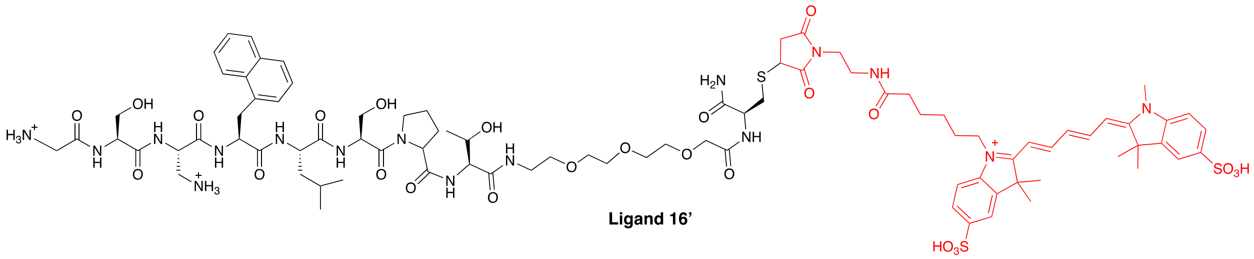

B)

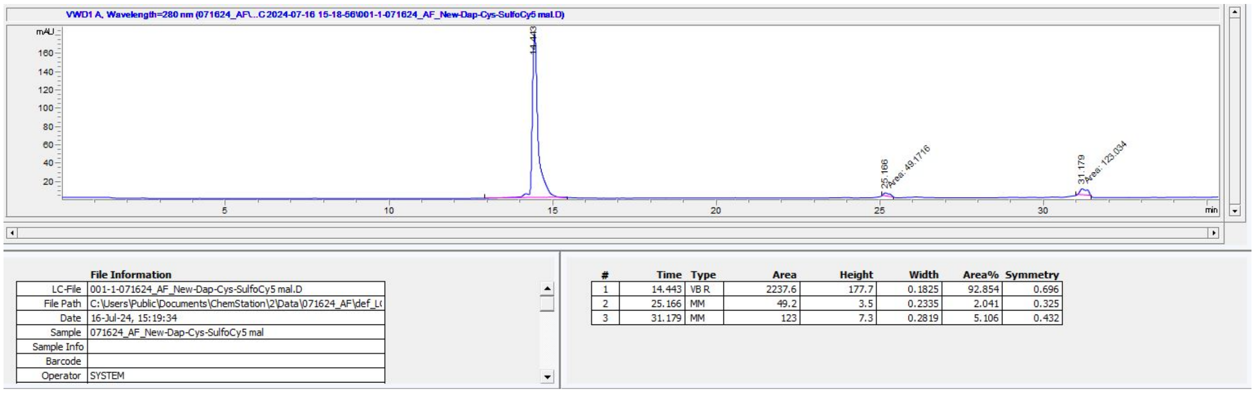

#	Time	Type	Area	Height	Width	Area%	Symmetry
1	14.443	VB R	2237.6	177.7	0.1825	92.854	0.696
2	25.166	MM	49.2	3.5	0.2335	2.041	0.325
3	31.179	MM	123	7.3	0.2819	5.106	0.432

C)

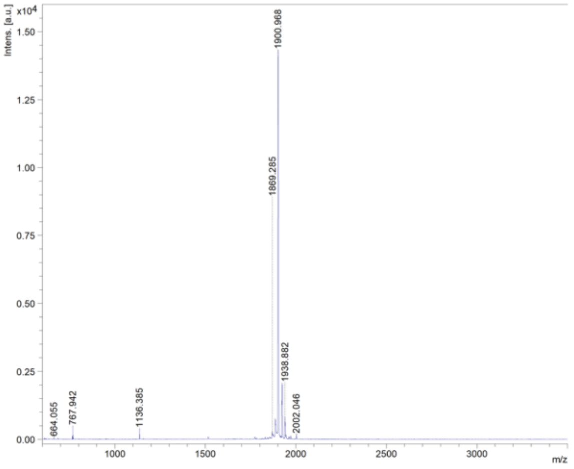

D)

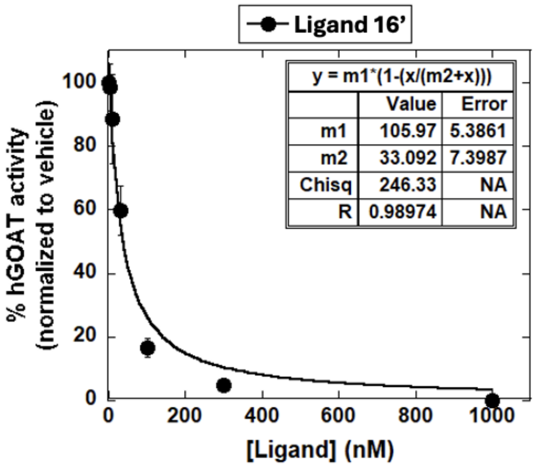

Figure S17. Characterization information for Ligand 16’. A) Chemical structure of Ligand **16’**. B) RP-HPLC chromatogram of Ligand **16’** with retention time at 14.4 minutes depicting purity. C) MALDI-TOF spectrum of purified Ligand **16’** with $[M+H]^+$ = 1900.968. D) IC_{50} value for Ligand **16’** against hGOAT (IC_{50}= 33.1 ± 7.4 nM). IC_{50} values against hGOAT represent the average of three independent trials. All protocols are reported in the Methods section.

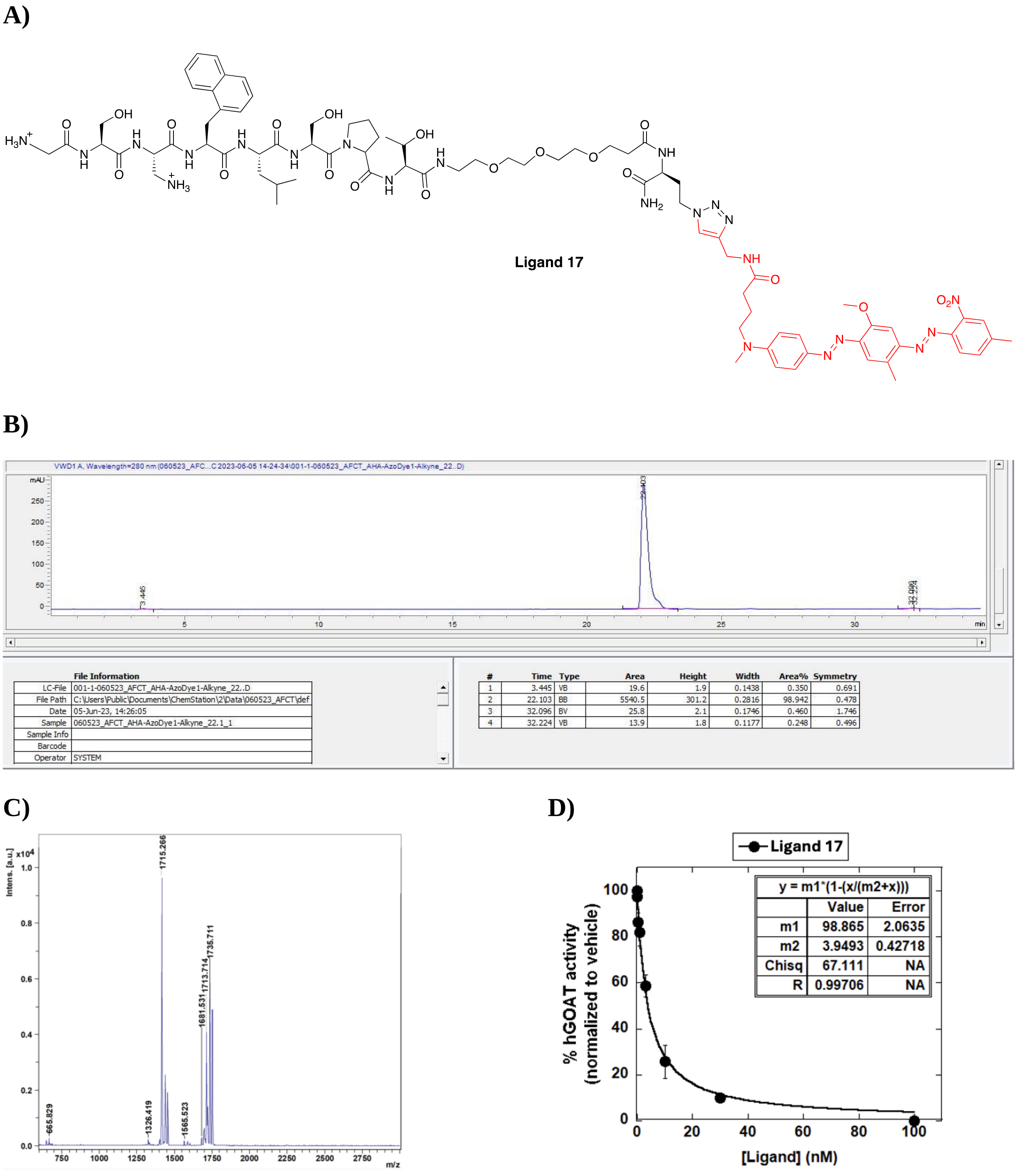

Figure S18. Characterization information for Ligand 17. A) Chemical structure of Ligand **17**. B) RP-HPLC chromatogram of Ligand **17** with retention time at 22.1 minutes depicting purity. C) MALDI-TOF spectrum of purified Ligand **17** with $[M+H]^+$ = 1713.714. D) IC_{50} value for Ligand **17** against hGOAT (IC_{50}= 3.9 ± 0.4 nM).. IC_{50} values against hGOAT represent the average of three independent trials. All protocols are reported in the Methods section.

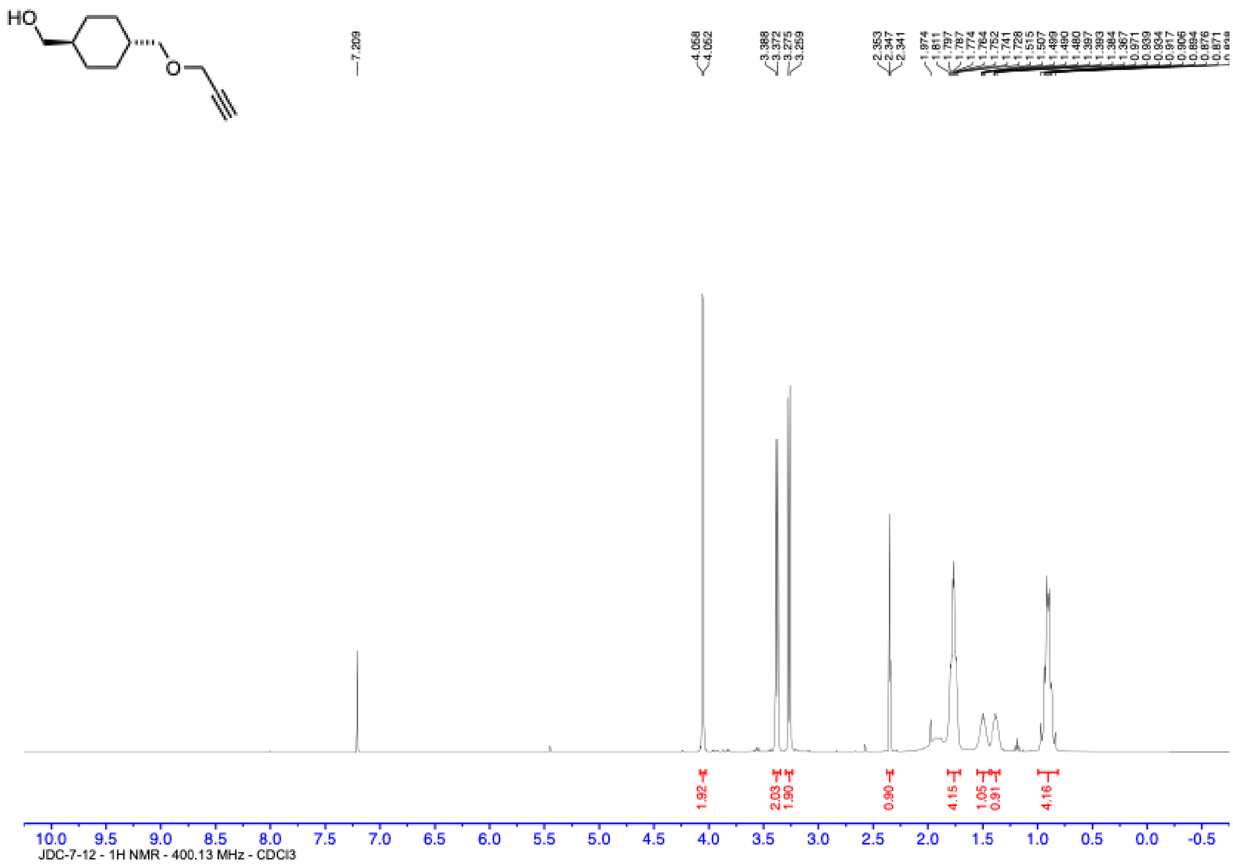

Figure S19. ^{1}H NMR spectrum for compound S1.

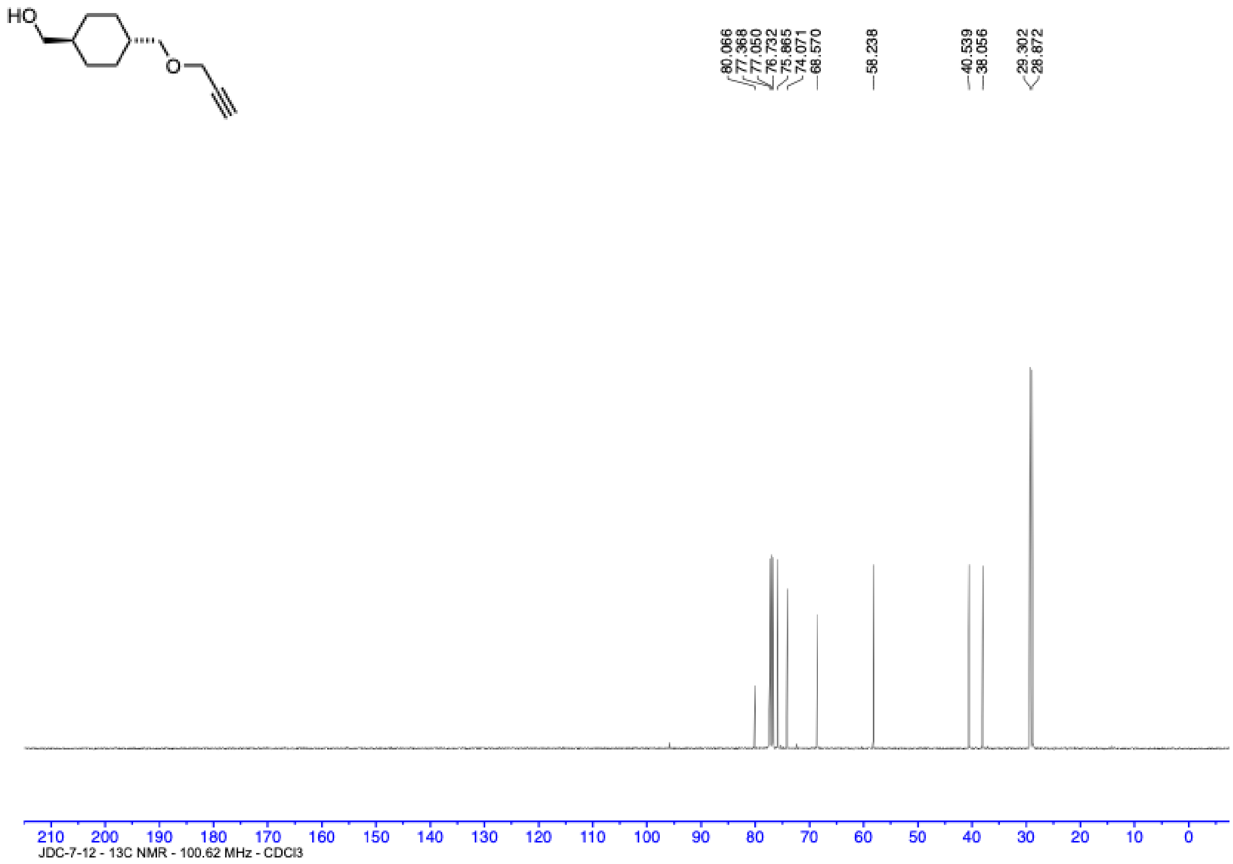

Figure S20. ^{13}C NMR spectrum for compound S1.

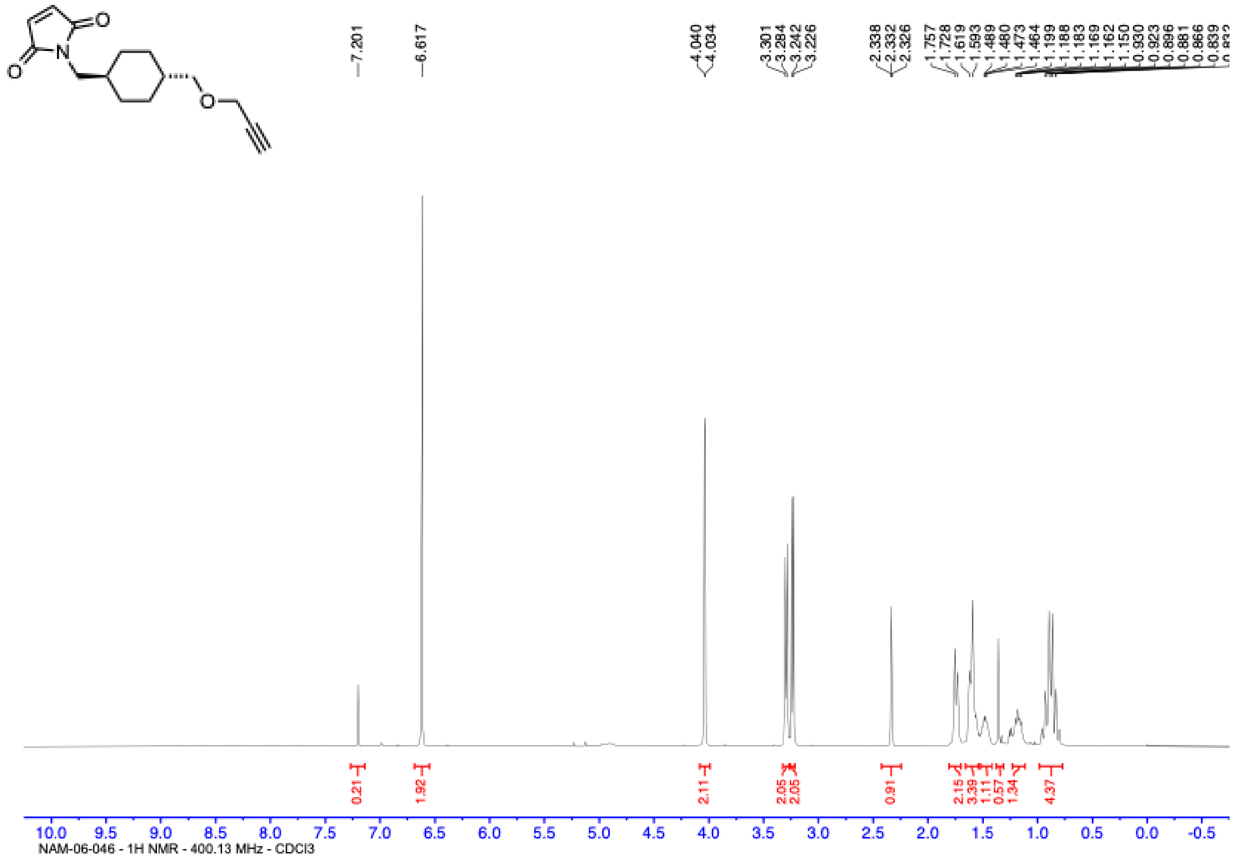

Figure S21. ^{1}H NMR spectrum for compound S2.

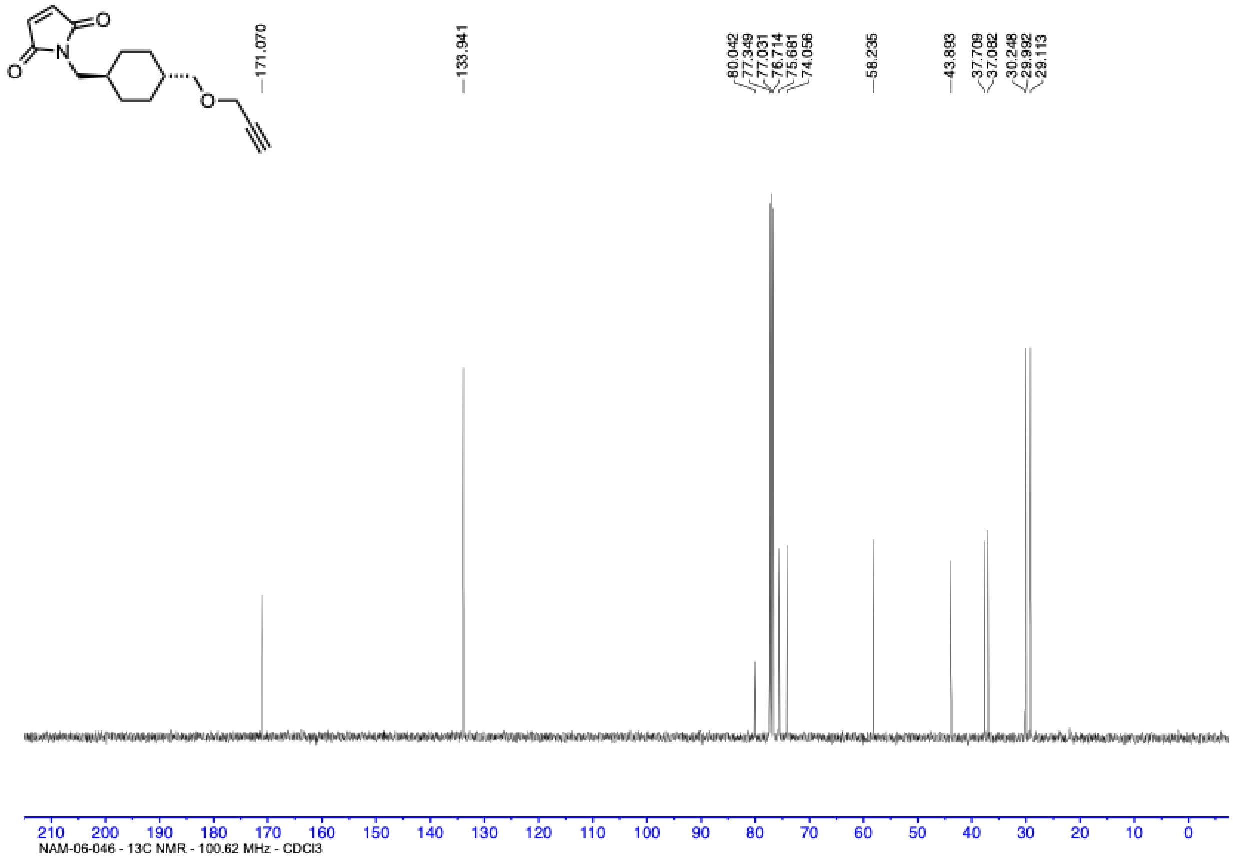

Figure S22. ^{13}C NMR spectrum for compound S2.

Supplementary References

(1) Kaufmann, T.; Wendeln, C.; Gokmen, M. T.; Rinnen, S.; Becker, M. M.; Arlinghaus, H. F.; Prez, F. D.; Ravoo, B. J. Chemically Orthogonal Trifunctional Janus Beads by Photochemical “Sandwich” Microcontact Printing. *Chem. Commun.* **2012**, *49* (1), 63–65. https://doi.org/10.1039/C2CC36483B.
